# Global variations, temporal trends, and socioeconomic inequalities in the burden of malignant brain and central nervous system tumors: a population-based systematic analysis of GLOBOCAN 2024

**DOI:** 10.1016/j.mmr.2026.100072

**Published:** 2026-10-06

**Authors:** Shao-Yuan Lei, Yue Wu, Hong-Jun Liu, Xiao-Feng Jia, Yan-Su Guo, Rong-Shou Zheng

**Affiliations:** a Evidence-Based Medicine Center, Xuanwu Hospital, Capital Medical University, Beijing 100053, China; b China National Health Development Research Center, Beijing 100044, China; c Beijing Geriatric Healthcare and Disease Prevention Center; Xuanwu Hospital, Capital Medical University, Beijing 100053, China; d Beijing Municipal Geriatric Medical Research Center, Beijing 100053, China; e Office for Cancer Registry, National Cancer Center/National Clinical Research Center for Cancer/Cancer Hospital, Chinese Academy of Medical Sciences and Peking Union Medical College, Beijing 100021, China

**Keywords:** Brain and central nervous system (CNS) tumors, Incidence, Mortality, Lifetime risk, Global cancer burden

## Abstract

**Background:**

Previous assessments of the burden of malignant brain and central nervous system (CNS) tumors have mainly focused on incidence and mortality rates, with less emphasis on lifetime risks. This study aimed to estimate the incidence, mortality, and lifetime risk of developing or dying from brain and CNS tumors and the impact of socioeconomic inequality across countries.

**Methods:**

Data on brain and CNS tumors were obtained from GLOBOCAN 2024 and the Cancer Incidence in Five Continents Plus (CI5plus) database, population and death data were sourced from World Population Prospects 2024. Lifetime risk estimation was conducted using the life table method adjusted for multiple primaries. We assessed trends in brain and CNS tumors incidence, lifetime risks, and histological subtypes during 2003 to 2017. The association between the human development index (HDI) and lifetime risks was assessed using generalized linear models.

**Results:**

In 2024, the age-standardized incidence and mortality rates for brain and CNS tumors were 3.37 and 2.51 per 100,000 population, respectively. The estimated global lifetime risk of developing or dying from brain and CNS tumors, from birth to death was 0.39% and 0.32%, respectively. Significant variations in lifetime risks were observed among countries and regions, as well as across different HDI levels. The lifetime risk of developing brain and CNS tumors among countries with a low, medium, high, and very high HDI was 0.16%, 0.19%, 0.45%, and 0.59%, respectively; the risks of dying from the disease in these categories were 0.14%, 0.17%, 0.34%, and 0.48%, respectively. Histological subtypes of brain and CNS tumors showed large discrepancies among age groups and countries. The most frequently diagnosed histological type was astrocytic tumors.

**Conclusions:**

We found significant heterogeneity in the incidence, mortality, and lifetime risk of brain and CNS tumors among geographic and HDI levels, which potentially reflects disparities in medical resource allocation and genetic exposures.

## 1 Background

Malignant brain and central nervous system (CNS) tumors are one of the most serious cancers globally, accounting for approximately 1.6% and 2.6% of all cancer cases and deaths worldwide in 2022, respectively [1]. Primary brain and CNS malignant tumors exhibit distinct histological and epidemiological profiles. According to the 2021 WHO Classification of Tumors of the CNS, gliomas constitute the most prevalent malignant subtype (75%–80% of cases), with glioblastoma representing the dominant histology and demonstrating the poorest prognosis. Meningiomas account for 20%–25% of all CNS tumors, predominantly affecting female patients [2]. Most subtypes of brain and CNS tumors have the characteristics of rapid onset, poor prognosis, and high relapse rates [3], [4], [5], with 5-year survival rates ranging from 20% to 40% in adults and 20% to 80% in children [6], leading to serious public health issues and a heavy economic burden. Mapping the epidemiological distribution of the brain and CNS tumor burden is essential for rational healthcare resource allocation, encompassing local and regional health systems to national policy frameworks.

Previous estimates of the burden of brain and CNS tumors have mainly focused on incidence and mortality by sex and subtype [7], [8], [9] in a specific region [10], [11], [12] or specific population [13]. Lifetime risk refers to the cumulative probability of being diagnosed with or dying from a disease at a specific point during the lifespan, assuming no competitive cause of death [14]. This is usually calculated by combining the number of new cases and deaths from brain and CNS tumors with the all-cause mortality of the entire population, which can reflect the epidemic of the disease to a certain extent. However, few studies have measured the lifetime risk of brain and CNS tumors [15]. Quantifying the probability of the lifetime risk of developing or dying from brain and CNS tumors will provide a novel perspective for developing precision prevention strategies. The human development index (HDI) serves as a composite indicator that highlights a country’s development level, incorporating indices such as education, life expectancy, and gross national income [16]. Disparities in HDI levels persist across regions and countries, and few studies have explored the association between HDI levels and the lifetime risks of brain and CNS tumors.

In this study, we aimed to estimate the effects of socioeconomic inequalities on the burden of brain and CNS tumors and temporal trends in the incidence, mortality, and lifetime risk of developing or dying from brain and CNS tumors across different populations at the national level, including the trend for different histological subtypes from 2003 to 2017. The present results will provide valuable guidance for planning global brain and CNS tumors.

## 2 Methods

### 2.1 Data sources

Data on the incidence and mortality of primary malignant brain and CNS tumors, coded as C70−72, according to the International Classification of Diseases, 10th edition, across different sexes and age groups (0−4, 5−9, …, 80−84, ≥85) in 2024 were sourced from the GLOBOCAN 2024 database (https://gco.iarc.who.int/today/) [17], recognized as the premier data source for cancer incidence and mortality worldwide. A total of 122 population-based cancer registries located in 44 countries covering 15 consecutive calendar years (2003–2017) of data were provided in the Cancer Incidence in Five Continents Plus (CI5plus) database, of which 23 registrations that reported detailed racial data were excluded. In addition, 24 registrations in 14 countries were further excluded due to national population coverage of less than 1 million, a country with only one cancer registration, a less than 5 cases of brain and CNS tumors in a certain year and an unknown histological subtype exceeding 75%. To analyze temporal trends, 75 population-based cancer registries reported continued data from 2003 to 2017 located in 30 countries were selected, and categorized by sex, age, and histological subtype. Comprehensive information on all-cause mortality and population data for different countries and regions in 2024 was obtained from the World Population Prospects 2024 (https://population.un.org/wpp/). Demographic factors such as population density and population living in urban areas were obtained from the United Nations Statistics (http://data.un.org/). We classified 186 countries according to 20 predefined geographic regions and categorized the countries according to the United Nations Development Programme’s HDI predefined levels in 2022 (very high ≥0.80, high 0.70−0.79, medium 0.57−0.69, and low <0.57) [16]. The HDI is a comprehensive indicator used to measure the average achievement in key dimensions of human development and reflects the well-being of countries according to three dimensions: health, education, and an acceptable standard of living. The levels of HDI classification for different countries are detailed in the **Additional file 1:** Table S1.

### 2.2 Statistical analysis

The lifetime risk was calculated using the life table method and adjusted for multiple primaries (AMP method) [14], [15], [18]. This method was adjusted for deaths caused by other non-brain and CNS tumors, and the possibility of multiple primary cancers was also considered. The frequency data per 5-year age group, including the number of new cases and deaths due to brain and CNS tumors, as well as population and all-cause deaths per 5-year age group in different countries and regions, were used to estimate lifetime risk of brain and CNS tumors at the population level by sex, HDI level, and age interval. The Poisson method was used to estimate the variances and calculate the 95% confidence interval (CI) of lifetime risk. Further details regarding the methods are provided in the **Additional file 2: Methods**. In this study, “lifetime risk” was defined as the cumulative probability over a specific period, such as from the age of 40, 50, 60, or 70 years old until death, and not necessarily the probability from birth to death. Sensitivity analyses were conducted to compare lifetime risks with cumulative risks, which is a common index of the cancer burden used to calculate cumulative risk without adjusting for other competing risks and life expectancy. We used a generalized linear model with a Poisson distribution and log link function to assess inequalities in human development. The model included the lifetime risk, age-standardized incidence rate (ASIR), and age-standardized mortality rate (ASMR) as the dependent variables, with HDI levels and their individual components as independent variables. Overdispersion was evaluated using the ratio of the Pearson chi-square statistic to its degrees of freedom. We reported risk ratios (*RR*s) and their 95% CI for the lifetime risk of developing or dying from brain and CNS tumors according to different HDI levels, based on unadjusted and multivariable-adjusted models, the latter of which included adjustments for population density and percentage of the population living in urban areas. Countries with continuous qualified data from 2003 to 2017 were included to analyze the temporal trends of brain and CNS tumors by histological subtype. The average annual percent change (AAPC) was estimated using the Joinpoint Regression Program (version 4.9.1, National Institutes of Health, Bethesda, MD, USA) [19]. To minimize the risk of reporting spurious trend changes over the period, all models were restricted to a maximum of 4 joinpoints (5-line segments). All data analyses were performed using SAS software (version 9.4, SAS Institute, Cary, NC, USA).

## 3 Results

### 3.1 Global incidence, mortality, and lifetime risks of developing or dying from brain and CNS tumors

Table 1 shows the ASIR, ASMR, and probability of developing or dying from brain and CNS tumors worldwide. The global ASIR and ASMR were 3.37 and 2.51 per 100,000 population, with a lifetime risk of developing or dying from brain and CNS tumors from birth to death of approximately 0.39% and 0.32% in 2024, respectively. Countries or regions with very high HDI had the highest ASIR and ASMR values (4.69 and 3.23 per 100,000 population, respectively) and the greatest lifetime risk (0.59% and 0.48%, respectively), while countries or regions with low HDI had the lowest ASIR (1.97 per 100,000 population) and lifetime risk (0.16% and 0.14%, respectively) of developing or dying from brain and CNS tumors. Among different regions, Northern Europe had the highest ASIR (5.63 per 100,000 population) and Eastern Europe had the highest ASMR (3.86 per 100,000 population). Australia/New Zealand had the highest lifetime risk of developing (0.80%) or dying (0.64%) from brain and CNS tumors. Micronesia/Polynesia had the lowest ASIR (0.66 per 100,000 population) and Melanesia had the lowest ASMR (0.48 per 100,000 population). Middle Africa and Micronesia/Polynesia had the lowest lifetime risk of developing (0.07%) or dying (0.07%) from brain and CNS tumors (Table 1).

**Table 1:** ASIR, ASMR, and lifetime risks of developing or dying from brain and CNS tumors in both sexes, 2024.

**Characteristics**	**ASIR (per 100,000 population)**	**Lifetime risk of developing brain and CNS tumors [% (95% CI)]**	**ASMR (per 100,000 population)**	**Lifetime risk of dying from brain and CNS tumors [% (95% CI)]**
World	3.37	0.39 (0.39–0.39)	2.51	0.32 (0.32–0.32)
HDI level				
Very high HDI	4.69	0.59 (0.58–0.59)	3.23	0.48 (0.48–0.48)
High HDI	3.80	0.45 (0.45–0.45)	2.62	0.34 (0.34–0.34)
Medium HDI	2.03	0.19 (0.19–0.19)	1.69	0.17 (0.17–0.17)
Low HDI	1.97	0.16 (0.16–0.16)	1.70	0.14 (0.14–0.14)
World region				
Australia/New Zealand	5.56	0.80 (0.77–0.83)	3.60	0.64 (0.61–0.67)
Caribbean	2.87	0.35 (0.33–0.37)	2.32	0.30 (0.28–0.32)
Central America	2.42	0.28 (0.27–0.29)	2.00	0.26 (0.25–0.27)
Eastern Africa	1.31	0.11 (0.11–0.12)	1.18	0.10 (0.10–0.11)
Eastern Asia	4.11	0.51 (0.51–0.52)	2.35	0.34 (0.34–0.35)
Eastern Europe	4.91	0.51 (0.50–0.51)	3.86	0.45 (0.44–0.45)
Melanesia	0.82	0.07 (0.05–0.09)	0.48	0.04 (0.03–0.06)
Micronesia/Polynesia	0.66	0.07 (0.01–0.15)	0.66	0.07 (0.01–0.14)
Middle Africa	1.02	0.07 (0.07–0.08)	0.92	0.07 (0.06–0.07)
Northern Africa	3.53	0.33 (0.33–0.34)	3.14	0.31 (0.30–0.31)
Northern America	5.11	0.62 (0.61–0.63)	3.10	0.50 (0.49–0.51)
Northern Europe	5.63	0.75 (0.74–0.77)	3.79	0.60 (0.59–0.62)
South America	4.07	0.52 (0.51–0.53)	3.24	0.46 (0.45–0.47)
South-Central Asia	2.41	0.23 (0.23–0.23)	1.99	0.20 (0.20–0.20)
South-Eastern Asia	2.18	0.22 (0.22–0.23)	1.87	0.20 (0.19–0.20)
Southern Africa	1.49	0.13 (0.12–0.14)	1.39	0.13 (0.12–0.14)
Southern Europe	5.62	0.79 (0.78–0.80)	3.85	0.64 (0.63–0.65)
Western Africa	1.18	0.09 (0.08–0.09)	1.09	0.08 (0.08–0.08)
Western Asia	4.31	0.50 (0.48–0.51)	3.56	0.43 (0.42–0.44)
Western Europe	5.16	0.70 (0.69–0.71)	3.61	0.57 (0.57–0.58)

ASIR. Age-standardized incidence rate; ASMR. Age-standardized mortality rate; HDI. Human development index; CNS. Central nervous system

Globally, the ASIR of brain and CNS tumors in women (3.04 per 100,000 population) was lower than that in men (3.73 per 100,000 population), and the lifetime risk of developing brain and CNS tumors during their lifetime was 0.40% in men and 0.38% in women. Similar to ASIR, the ASMR of brain and CNS tumors was also lower in women (2.11 per 100,000 women) than in men (2.93 per 100,000 men). The lifetime risk of dying from brain and CNS tumors was 0.30% in women and 0.34% in men (**Additional file 1:** Tables S2, S3). Brain and CNS tumors accounted for a high proportion of the incidence and mortality in children and adolescents (aged <20 years), accounting for more than approximately 11% of cases and 17% of deaths in those under 15 years, while in adolescents (aged 15–19 years), the proportions were slightly lower, at 7.73% and 13.21%, respectively. The number of new cases and deaths due to brain and CNS tumors in different age groups decreased with increasing age. In the adult population aged ≥20 years, the proportion of new cases and deaths showed a gradually decreasing trend with age, falling below 5% after the ages of 25 and 40, respectively (**Additional file 2:** Fig. S1).

At the country level, Montenegro (8.20 and 5.48 per 100,000 population), North Macedonia (7.48 and 6.05 per 100,000 population), and Bulgaria (6.96 and 5.28 per 100,000 population) had the highest ASIR and ASMR. Greece (0.99%), Norway (0.98%) and Montenegro (0.95%) showed the highest lifetime risk of developing brain and CNS tumors and North Macedonia (0.87%), Montenegro (0.84%) and Greece (0.83%) had the highest lifetime risk of dying from brain and CNS tumors. Fiji, Cameroon, and Republic of the Congo had the lowest ASIR and ASMR values, with a lifetime risk of developing or dying from brain and CNS tumors only 0.02% (**Additional file 1:** Tables S4-S7**; Additional file 2:** Fig. S2). Generally, the age specific incidence rate increased with age; however, if a tumor has not been diagnosed before a certain age, the risk of being diagnosed with a tumor in the rest of the lifetime decreased gradually. The accrued risk of developing or dying from brain and CNS tumors before age 40 years was relatively low, with a risk of 0.34% for incidence in both sexes (0.33% in women and 0.34% in men) and 0.29% for mortality (0.27% in women and 0.30% in men). However, individuals who had not developed a brain and CNS tumors by age 70 years, showed a probability of 0.16% (0.17% in women, 0.15% in men) for incidence and 0.15% (0.15% in women, 0.14% in men) for mortality over the remaining lifespan (Fig. 1; **Additional file 1:** Tables S4-S9). There were large heterogeneities in lifetime risks among countries. More detailed results are provided in **Additional file 2:** Figs. S3-S8.

**Fig. 1:**
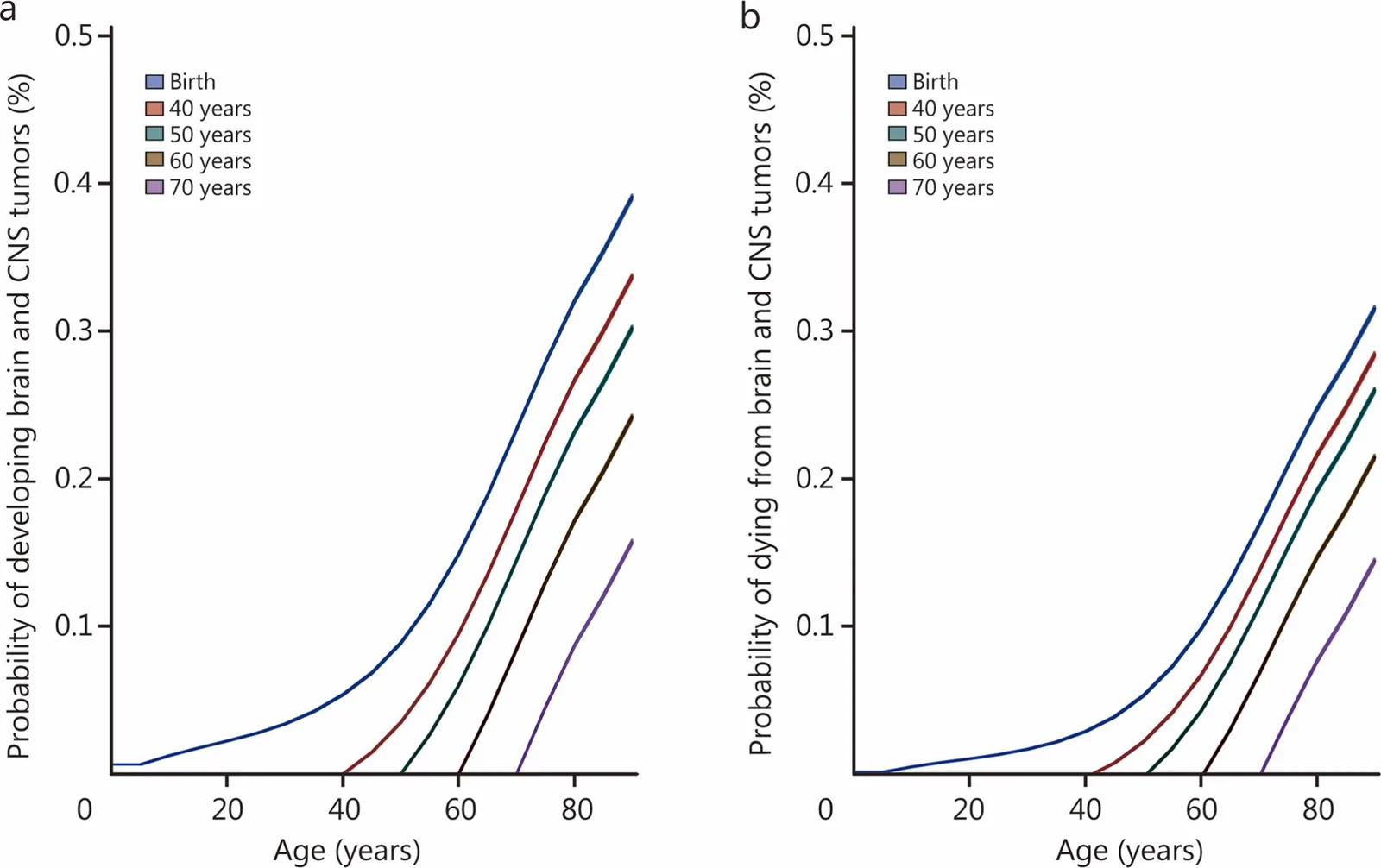
Lifetime risks of developing or dying from brain and CNS tumor within selected age intervals in 2024, both sexes. **a** Developing brain and CNS tumors. **b** Dying from brain and CNS tumors. Curves are represented for base line ages at birth, 40, 50, 60, and 70 years of brain tumor free life. Risks until death represent lifetime risk from the base line age. CNS. Central nervous system.

### 3.2 Comparisons of national life expectancy, cumulative risk, and lifetime risk

With an increased proportion of elderly people globally, we observed significant differences between the cumulative risk (over 0–74 years) and lifetime risk due to variations in life expectancy and competing death risk. We compared those two indicators and found significant differences in most populations (**Additional file 2:** Fig. S9). Among 88 countries or regions where the population had a life expectancy of ≥75 years of age, 93 countries had a higher lifetime risk than cumulative risk of developing brain and CNS tumors during 0–74 years. Of the 99 countries or regions with a population life expectancy <75 years of age, 70 countries showed a higher cumulative risk (over 0–74 years) (**Additional file 1:** Table S10; **Additional file 2:** Fig. S9). In contrast to the lifetime risk of developing brain and CNS tumors, 7 countries or regions presented a lower lifetime risk of dying compared with cumulative risk (age 0–74 years) in 88 countries or regions with a life expectancy ≥75 years. Twenty of the 99 countries or regions with a population life expectancy <75 years of age exhibited a higher lifetime risk of death due to brain and CNS tumors, in comparison with 99 countries having a life expectancy <75 years (**Additional file 1:** Table S10; **Additional file 2:** Figs. S10-S12).

### 3.3 Trends in the incidence and lifetime risk of developing brain and CNS tumors from 2003 to 2017

From 2003 to 2017, 9 selected countries showed upward trends in ASIR and 13 countries showed upward trends in lifetime risk. The largest increases occurred in Argentina, with an AAPC of 5.28% for ASIR and 7.13% for lifetime risk, Croatia had the lowest decreasing trends, with an AAPC of –2.32% for ASIR and –2.43% for lifetime risk (Fig. 2**; Additional file 2:** Figs. S13-S15). The changes in incidence varied across countries according to age group (**Additional file 2:** Fig. S16). The incidence among children and adolescents (aged 0−19 years) remained stable in most selected countries except Lithuania, Australia, and Croatia. The incidence in the early-onset population (aged 20−49 years) showed marked heterogeneities, with countries in Argentina, Japan, Italy, and Ireland showing increasing trends, and Iceland, Poland, the USA, China, and Croatia exhibiting decreasing trends (**Additional file 2:** Fig. S17). The incidence among children and adolescents at the global-scale remained stable; however, the incidence showed increasing trends in young adults aged 25−35 years and those aged ≥75 years during 2003−2017. Notably, there was a downward trend in incidence among adults aged 55−59 years.

**Fig. 2:**
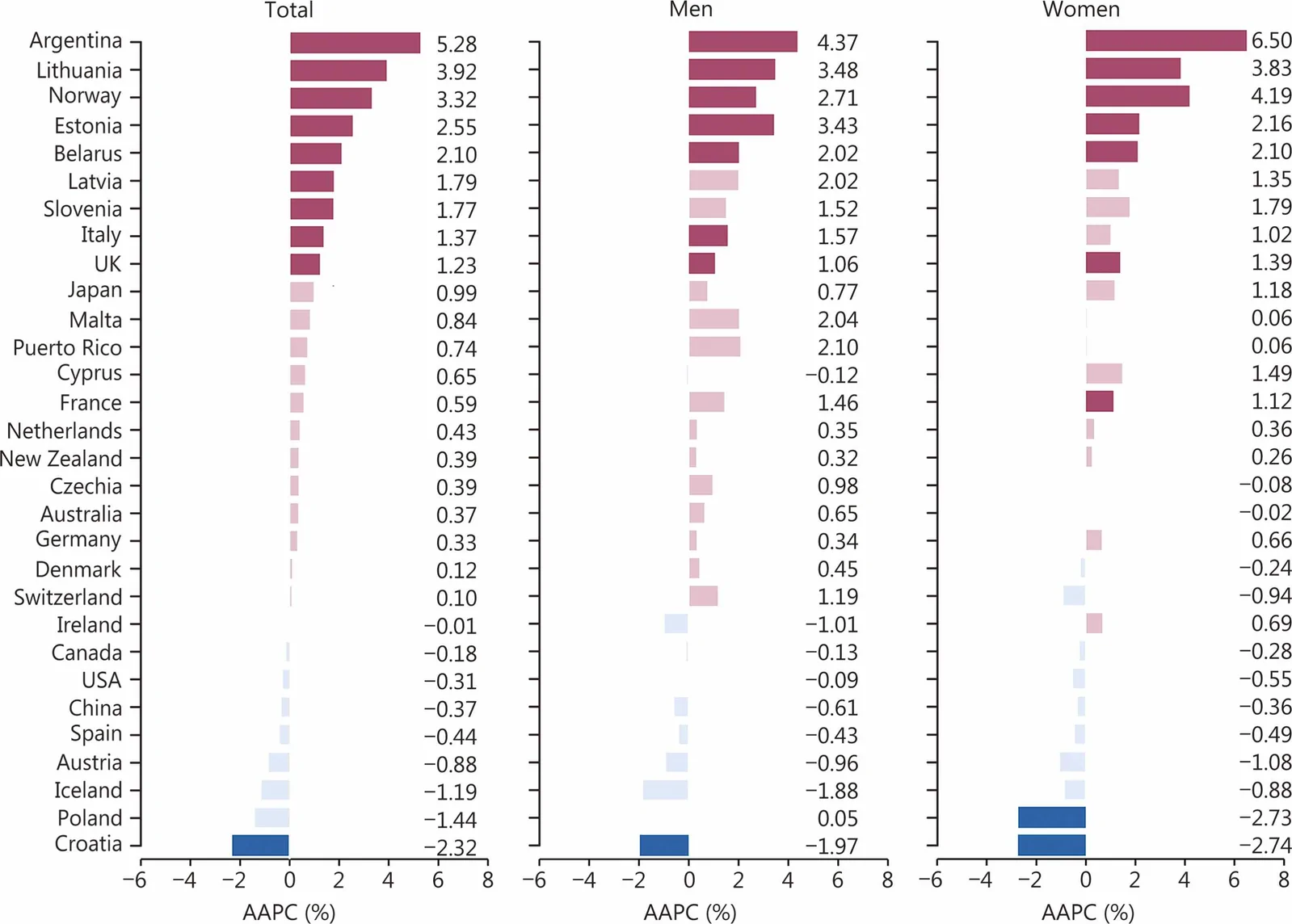
The average annual percent change (AAPC) in age-standardized incidence rate of brain and CNS tumors by country, 2003−2017. Dark red bars represent countries with a statistically significant increasing trend (AAPC>0, *P*<0.05). Light red bars represent countries with an increasing trend that is not statistically significant (AAPC>0, *P*≥0.05). Dark blue bars represent countries with a statistically significant decreasing trend (AAPC<0, *P*<0.05). Light blue bars represent the countries with a decreasing trend that is not statistically significant (AAPC<0, *P*≥0.05). CNS. Central nervous system.

We observed large discrepancies among age, country, and period for different histological subtypes of brain and CNS tumor. Astrocytic tumors were the most frequently diagnosed type among both men and women in most of the 30 selected countries, followed by meninges and gliomas of uncertain origin (Fig. 3; **Additional file 2:** Figs. S18, S19). Considerable heterogeneity was observed among different histological subtypes of brain and CNS tumors between children and adults. The proportion of astrocytic tumors was relatively low, whereas, the proportion of medulloblastoma and gliomas of uncertain origin was much higher in children and adolescents (aged <20 years) than that in adults (**Additional file 2:** Fig. S20). Similar patterns among histological subtypes were observed in men and women (**Additional file 2:** Figs. S21, S22). Among the 9 histological subtypes, astrocytic tumor, ependymal tumor, and medulloblastomas showed upward trends, while other embryonal tumors, oligodendroglial tumors, mixed gliomas, and other tumors with specified morphology showed downward trends during 2003–2017 (**Additional file 2:** Figs. S23, S24).

**Fig. 3:**
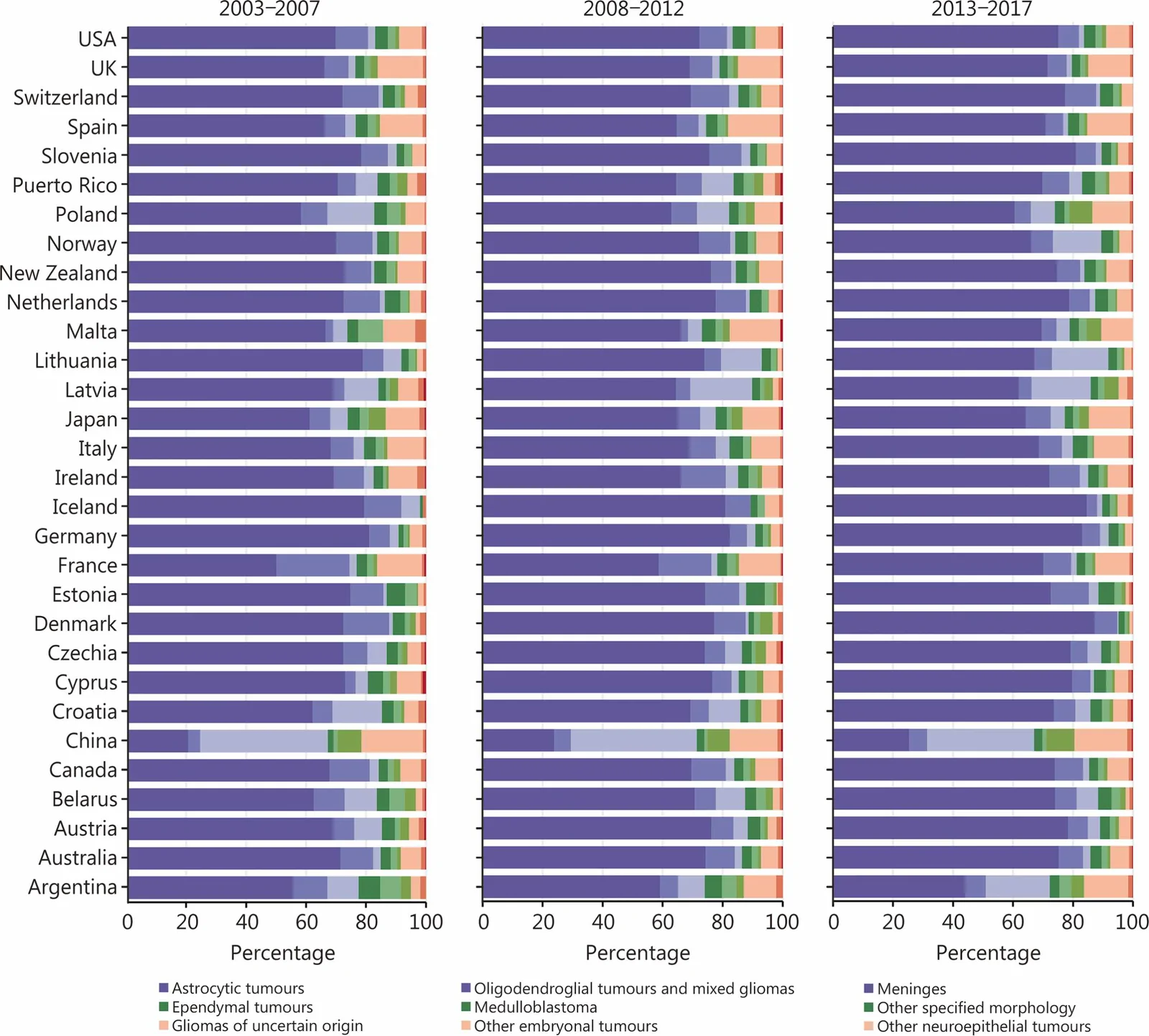
The percentage of brain and CNS tumor cases by subtype from 2003 to 2017 in both sexes. CNS. Central nervous system.

### 3.4 Effects of socioeconomic inequalities on lifetime risks of developing or dying from brain and CNS tumors

In the multivariable adjusted model, we observed a lower lifetime risk of developing brain and CNS tumors in high HDI levels (*RR*=0.62, 95% CI 0.49–0.78), medium HDI levels (*RR*=0.38, 95% CI 0.27–0.53) and low HDI levels (*RR*=0.22, 95% CI 0.15–0.33) in both sexes. Similar to the risk of developing brain and CNS tumors, a lower risk of dying was observed with high, medium, and low HDI levels with the corresponding *RR* values of 0.62 (95% CI 0.50–0.78), 0.40 (95% CI 0.29–0.55) and 0.23 (95% CI 0.16–0.34) for both sexes, respectively. The same trend was observed in both men and women for incidence and mortality risk with different HDI levels **(**Fig. 4**; Additional file 2:** Figs. S25, S26**)**. To better clarify the effects of socioeconomic inequalities on the developing or dying from lifetime risks of brain and CNS tumors, we performed a further analysis to examine the association among mean years of education, gross national income per capita, universal health coverage (UHC) index. Lower mean years of schooling were associated with significantly reduced developing risks in men (low HDI: *RR*=0.32, 95% CI 0.10–0.98). Similarly, lower gross national income per capita and lower UHC index were significantly associated with decreased lifetime risks of developing and dying from brain and CNS tumors. The detailed results were provided in **Additional file 2:** Figs. S27-S32.

**Fig. 4:**
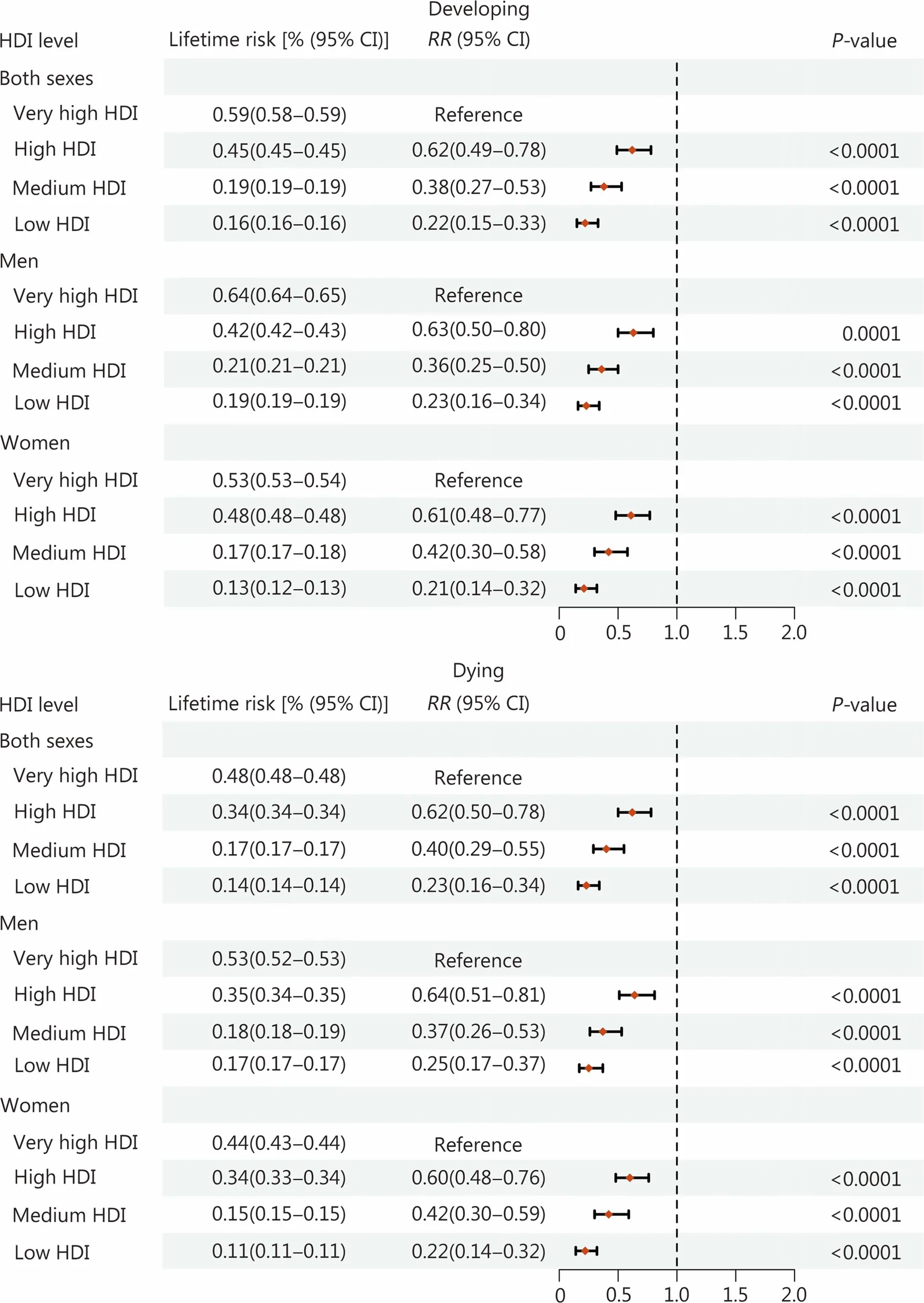
The association between human development index (HDI) and lifetime risk of brain and CNS tumors in 186 countries and territories, 2024. Multivariable models were adjusted for Current Health Expenditure (CHE) as a percentage of Gross Domestic Product (GDP), CHE per capita in United States Dollar (USD), population density, and percentage of population living in urban areas. *P*<0.05 means statistical significance. *RR*. Risk ratio; CI. Confidence interval; CNS. Central nervous system.

## 4 Discussion

We conducted a comprehensive analysis to delineate the distribution of incidence and mortality rates of brain and CNS tumors, estimated the lifetime risk, analyzed the socioeconomic disparities influencing the likelihood of developing or dying from brain and CNS tumors in 2024, and depicted the temporal trends of incidence, mortality, and lifetime risk for brain and CNS tumors according to age, sex, and histological subtype from 2003 to 2017. The ASIR and ASMR showed large variability across regions and countries, with disparities of approximately 5-fold between the highest and lowest-ranking nations. The pattern of brain and CNS tumors in children and adolescents was significantly different from those in adults, with relatively low rates for incidence (1.44%, 293,231/20,353,743) and mortality (2.39%, 231,320/9,660,241) in adults aged ≥20 years and above. However, the rates were relatively high in children and adolescents for incidence (10.92%, 30,864/282,698) and mortality (17.25%, 17,644/102,266). Globally, an estimated 4 in 1000 individuals were projected to develop or die from brain and CNS tumors during their lifetime among both men and women, and the risks increased with HDI levels. Using countries with the highest HDI levels as reference, we identified a lower risk of developing or dying from brain and CNS tumors in areas with low HDI levels. From 2003 to 2017, the temporal trends showed large discrepancies among countries, where the most frequent histological type was astrocytic tumors among the 30 selected countries.

Our estimation revealed the burden of brain and CNS tumors in multiple dimensions. Significant disparities were found globally, with very high risks and ASIR observed in Europe and Australia/New Zealand, whereas the lowest risks were found in most African countries. A positive association was observed between HDI level, ASIR, and lifetime risk of developing brain and CNS tumors. These results were in line with the findings of other studies that quantified the ASIR of brain and CNS tumors [9], [20], [21]. The variations in ASIR and risks of brain and CNS tumors during the lifespan probably reflect comprehensive factors, including disparities in access to healthcare, genetic susceptibility, and environmental exposures [11], [22], [23], [24], [25], [26]. The lower lifetime risk in countries with low HDI levels may be because these countries have fewer health resources, including for diagnosing brain and CNS tumors and access to medical care, resulting in underestimation of the true number of cases. Furthermore, countries with fewer resources may not have appropriate registry systems to track cancer rates, which may result in underreporting. Additionally, in low HDI countries, such as those in Africa, the lower lifetime risk of brain and CNS tumors is typically associated with the high competing risks of death from infectious diseases. Our results also indicated that both the life expectancy and lifetime risk of developing brain and CNS tumors increased with HDI levels (**Additional file 2:** Figs. S33, S34).

Heterogeneity could not be exclusively attributed to the accessibility of health resources; broader differences in genetic predisposition also play an important role. A previous study showed that the incidence rate of brain and CNS tumors, especially glioblastoma, was 2.5-fold higher in non-Hispanic White individuals than in Asian or Pacific Islander population [11]. Notably, environmental exposure is also a significant factor in disease onset, as a proven risk factor, ionizing radiation has been shown to lead to brain and CNS tumors [27], [28]. Other exposures, such as mobile phone usage and viral infections, have no direct evidence to date linking them with these tumors [29], [30], [31]. Our study further indicated that the age-standardized incidence and age-conditional probability of developing brain and CNS tumors were higher in men than in women, which is consistent with studies from the United States and Japan [11], [32]. Differences in hormones and genetic features may account for sex-based disparities [33]. Notably, no population-based screening program is currently available for the early detection of these tumors. Future studies are needed to thoroughly explore the etiology and develop simpler diagnostic technologies for the prevention and management of brain and CNS tumors. The accessibility of medical resources, such as computed tomography (CT) and magnetic resonance imaging (MRI), may increase the burden of brain and CNS tumors. A possible reason for this may be that the diagnosis of brain and CNS tumors relies heavily on these advanced techniques, and increasing access to CT and MRI could lead to a relative increase in the number of diagnosed brain and CNS tumors. Additionally, CT is an indispensable medical imaging test that is widely performed in diagnosis, but that exposes patients to ionizing radiation, which is known to be associated with increased cancer risk, as noted above [28], [34], [35], [36]. Several large cohort studies have shown that childhood exposure to CT is associated with an increased risk of hematologic malignant neoplasms and brain cancer [35], [36].

Similar to the ASIR and lifetime risk of developing brain and CNS tumors, we observe significant variations in the ASMR and lifetime risk of mortality globally. The ASMR ranged from 0.48 per 100, 000 in Melanesia to 3.86 per 100,000 population in Eastern Europe, which is consistent with the findings of two previous studies [9], [20]. The highest probability of a person dying from brain and CNS tumors during their lifespan occurred in Europe and some Asian countries, whereas the lowest risks were observed in most African countries. The 5-year survival rate for the disease is low, especially for glioblastoma [6], and the optimal treatment depends on advanced health-care resources, neurosurgical services, intensive care units, and specialized radiation or chemotherapy. The relatively difficult accessibility in many areas may explain the heterogeneity in ASMR values [18], [37], [38]. Additionally, in most countries and regions, the incidence rate is relatively low for brain and CNS tumors, as compared with those for other cancers. If a country or region lacks medical resources, the attention paid to those tumors will be further reduced, which may lead to lower incidence rates reported in these areas. We also observed that the risk of dying from brain and CNS tumors decreased with age, and the residual risk varied greatly among different countries and age groups. This may be because survival to an older age results in a lower lifetime risk during the remainder of the lifespan. In general, patients with these tumors may have poor prognosis and severe disability. The lifetime risk of death could provide important information for resource reallocation and strategic prevention on a global-scale.

The temporal trend in the burden of brain and CNS tumors revealed that the incidence rate and lifetime risk had an upward trend in most countries. With the improvement of cancer registration systems, traditional paper-based case cards have been substituted with electronic data collection, which enables more comprehensive and accurate acquisition of patient treatment information. Additionally, the electronic data exchange has reduced missed diagnoses and insufficient registration, which may lead to the identification of more cases. The mortality-to-incidence ratio of brain and CNS tumors was relatively higher in low/medium HDI areas than those in high HDI areas, indicating the possibility of fewer treatment opportunities and delayed diagnosis.

The lifetime risk found in this study reflects the risk of developing or dying from brain and CNS tumors over an individual’s actual lifespan, which is a crucial indicator for assessing population-level cancer risk across various age groups, considering the life expectancy of the population and competing risks of death from other diseases [18]. In sensitivity analysis, assuming that the incidence and mortality rates of brain and CNS tumors remained unchanged globally, but if the life expectancy reached the level of Japan (life expectancy: 84.85 years), which means the age-specific mortality rate for all causes of death was the same as Japan, the absolute lifetime risk of developing brain and CNS tumors was estimated to increase from 0.39% to 0.53%, corresponding to a relative increase of approximately 34.96%. Alternatively, if the life expectancy reached the level of Nigeria (life expectancy: 54.63 years), which means the age-specific mortality rate for all causes of death was the same as Nigeria, the absolute lifetime risk may be reduced from 0.39% to 0.22%, corresponding to a relative decrease of approximately 43.09%. Therefore, accurate assessment of the disease burden can support optimal cancer prevention and control policies in a country or region; however, an inaccurate burden estimation may result in misallocation of public health resources. Brain and CNS tumors exhibit a bimodal age distribution with distinct molecular drivers. Overall, as age increases, the cumulative risk of onset and mortality gradually increases; however, the probability of developing or dying of brain and CNS tumors in the elderly population remains high. These age-specific patterns suggest the need for customized risk stratification and precise treatment in different age groups for prevention and control. Pediatric cases are dominated by embryonal tumors and gliomas, often driven by developmental pathway alterations like Sonic hedgehog or Wingless-related integration site signaling, which confer survival advantages in specific subtypes. By contrast, older adults have a predominance of glioblastoma and meningiomas, characterized by accumulated genomic damage such as telomerase reverse transcriptase (*TERT)* promoter mutations and epidermal growth factor receptor (EGFR) amplification in isocitrate dehydrogenase (IDH) wildtype glioblastoma, contributing to poor prognosis [2].

The main strength of this study is that we used the most recent available cancer data for 2024 to estimate for the first time the lifetime probability of developing or dying from brain and CNS tumors. Our findings can provide an up-to-date perspective on the global burden of brain and CNS tumors. Lifetime risks provide a more intuitive and comparable measure of the disease burden by adjusting for multiple factors, including competing risks of death from all causes, life expectancy, socioeconomic development, and population ageing. This approach allows for a more direct measurement of the burden of brain and CNS tumors than traditional indexes [39], [40]. We also examined potential disparities in the risk of developing or dying from brain and CNS tumors among individuals from diverse socioeconomic backgrounds. This information holds great importance in guiding the allocation of medical resources, establishing appropriate priorities for the prevention and control of brain tumors and improving public health policies. This study has several limitations. First, the data on brain and CNS tumors were obtained based on the GLOBOCAN database, which relies on high-quality cancer registry data and vital registration systems. However, data availability is limited in some under-developed countries, and the mortality statistics only covered approximately one-third of the world’s population [41]. The incidence data in 33 countries were estimated based on the rates in neighboring countries or registrations in the same area, accounting for approximately 7.7% of the global population and 3.4% of the estimated global incidence of brain and CNS tumors. Given the limited coverage of population-based cancer registration data, particularly in low- and middle-income countries, the results for these countries require further validation. Second, trend analysis relied on the CI5plus database, which is subject to a certain reporting lag. Nonetheless, this database includes the highest-quality cancer surveillance data currently available, together with histological subtype information. Third, life-threatening conditions can vary by subtype; however, we were unable to estimate the lifetime risk according to histology owing to the small number of cases. Fourth, the lifetime risk was estimated based solely on the age-specific incidence and mortality in 2024, without considering potential future changes in rates. Fifth, it is important to note that this was a population-based ecological study. Consequently, the findings reflect lifetime risks of brain and CNS tumors at the group level (global, regional, national) and cannot be extrapolated to individual-level risk. Future research using individual-level study designs, such as cohort studies, is needed to assess risk at the individual level.

## 5 Conclusions

In this study, we comprehensively described the age-standardized incidence and mortality rates of brain and CNS tumors and, estimated the lifetime risks adjusting for competing risks of death, life expectancy, and other factors at the global, regional, and country levels by sex and age. Globally, the estimated lifetime probability of developing or dying from brain and CNS tumors for both men and women was approximately 4 in 1000 individuals, with significant variations across different regions worldwide in 2024. Furthermore, lifetime risks were observed to be higher in men than in women for brain and CNS tumors, and these risks decreased with age. There is a contradictory interaction between lifespan and lifetime risk; an increase in life expectancy leads to an increased risk of developing cancer in the population; however, an increased risk indicates accelerated individual death, which may reduce the life expectancy. This two-way relationship can be modified through adopting healthy lifestyles and prevention strategies such as precision medicine. Overall, our analysis provides a comprehensive understanding of the risks of brain and CNS tumors in different geographic and economic contexts, which will contribute to optimizing resource allocation and health strategy planning on a global scale, as well as combating brain tumors and addressing health inequalities that exist throughout the entire cancer process.

## Abbreviations

AAPC: Average annual percent change

AMP: Adjusted for multiple primaries

ASIR: Age-standardized incidence rate

ASMR: Age-standardized mortality rate

CNS: Central nervous system

CI: Confidence interval

CI5plus: Cancer Incidence in Five Continents Plus

CT: Computed tomography

EGFR: Epidermal growth factor receptor

HDI: Human development index

MRI: Magnetic resonance imaging

WHO: World Health Organization

TERT: Telomerase reverse transcriptase

IDH: Isocitrate dehydrogenase

## Declarations

### Ethics approval and consent to participate

This study does not involve any identifiable personal or medical data from living individuals. Accordingly, the Ethical Committee of the National Cancer Center/Cancer Hospital, Chinese Academy of Medical Sciences, and Peking Union Medical College waived the need for ethical approval or informed consent for this study.

## Funding

This work was supported by the Capital’s Funds for Health Improvement and Research (CFH2024-2G-40214), the National Key Research and Development Program (2025YFC2708601), the Chinese Academy of Medical Sciences (CAMS) Innovation Fund for Medical Sciences (2021-I2M-1-011), the Project for Innovation and Development of Beijing Municipal Geriatric Medical Research Center (11000023T000002036320), and the Yingcai Training Program (YC20220128).

## Data Availability

All the data used in this study were obtained from a public database. Cancer data are available online at https://gco.iarc.fr/today/. The population, all-cause mortality and life expectancy data are available at https://population.un.org/wpp/. Another request for the full dataset is available from the corresponding author.

## Footnotes

Appendix A Supplementary data associated with this article can be found in the online version at doi:10.1016/j.mmr.2026.100072.
