# Supplementary material for: Global variations, temporal trends, and socioeconomic inequalities in the burden of malignant brain and central nervous system tumors: a population-based systematic analysis of GLOBOCAN 2024
Source: Mil Med Res. 2026 Oct 6;13(1):100072. doi: 10.1016/j.mmr.2026.100072 (PMC13676099; doi:10.1016/j.mmr.2026.100072)
Supplement: Supplementary file 1 [file mmc2.pdf]

Methods

A short introduction about the methods of lifetime risk of developing or dying from brain and central nervous system (CNS) tumors

The lifetime risk of cancer is an estimate of a person's chances of being diagnosed with cancer at some point during their life. Lifetime risk estimates are usually expressed as the odds of developing cancer ("1 in X"), as a percentage, or as the number of people diagnosed per 100 people.

Here we describe briefly introduction about the methods of lifetime risk of developing or dying from brain and CNS tumors as follows:

Cumulative rate

The cumulative rate can be calculated by the formula:

$$\text{Cumulative rate}=\sum_{i=1}^{A} W_i R_i$$

A denotes the age-band, usually use 74 for cumulative rate calculation; W_i denotes the width of the ith age interval; R_i denotes the age-specific incidence rate (ASIR).

Cumulative risk

The cumulative risk can be calculated by the formula:

$$\text{Cumulate risk}=1-e^{-\text{cumulative rate}}$$

Average annual percent change (AAPC)

The AAPC over any fixed interval is calculated using a weighted average of the slope coefficients of the underlying Joinpoint regression model with the weights equal to the length of each segment over the interval. The final step of the calculation transforms the weighted average of slope coefficients to an annual percent change. The formula for the calculation of AAPC is as follows:

$$\text{AAPC}=\left[\exp\left(\frac{\sum w_i b_i}{\sum w_i}\right)\text{-1}\right]\times 100$$

where for a fixed interval: i denotes the segments in the desired range of years; b_i denotes the slope coefficient; w_i denotes the length of each segment in the range of years.

The "adjusted for multiple primaries (AMP)" method

The lifetime risk of cancer is an important measure of the population-level risk of cancer across different age intervals and aids long-term health systems planning. Lifetime risk measures the cumulative probability of an individual being diagnosed with cancer or dying with cancer during their lifetime. Compared with the traditional incidence rate or mortality rate, the indicator of the lifetime risk of the population can provide another measurement standard to assess the cancer burden for people in different countries or regions around the world. This indicator can also assess the probability of cancer in the remaining life of an individual. In theory, the method to obtain the lifetime cancer risk of a population should be to track and observe specific populations from birth to death, collect all cancer information for the calculation, but in practice, it is very difficult to achieve unless population-based cancer registration work can last for a long enough period to cover the whole stages of a population from birth to death. The traditional method for assessing the lifetime cancer risk of a population is to use the cumulative risk between the ages of 0−74 to approximate reflect the lifetime risk of the population. This is a very simple and clear calculation and principle, but the results may also have significant deviations due to life expectancy or competitive mortality risk was not taken into consideration. In countries or regions with higher life expectancy, the cumulative risk between the ages of 0−74 may underestimate the lifetime cancer risk; in countries or regions with lower life expectancy, due to the higher risk of death caused by competition, using the cumulative risk from 0 to 74 years old may overestimate the lifetime risk of cancer.

The AMP method was used to calculate the lifetime risk, which takes into account deaths from other causes, and corrects for the inclusion of multiple primary cancers in the incidence rates. Cancer incidence, cancer mortality and all-cause mortality rates by 5-year age group were used to estimate the lifetime risk of cancer by sex at different age

spans which represents the probability of being diagnosed with cancer from that age onwards. The AMP method has been introduced in detail in previous studies [1-3], and here is a brief introduction. Using all-cause mortality and cancer incidence, mortality, we calculated the lifetime risk by 5-year age groups:

$$\mathrm{P_{AMP}}=\int_0^{\infty}\hat{\lambda}_c(a)\hat{S}_0^*(a)\,da=\sum_{i=1}^{f}\frac{R_i}{R_i+M_i\text{-}D_i}\hat{S}_0^*(a_i)\times\left(1\text{-exp}\left(\text{-}\frac{w_i}{N_i}(R_i+M_i\text{-}D_i)\right)\right)$$

where for age group: P_{AMP} denotes the probability of being diagnosed with brain and CNS tumors; M_i denotes the annual number of deaths (all-cause mortality); D_i denotes the annual number of brain and CNS tumors deaths (brain and CNS tumors mortality). When estimating the risk of developing brain and CNS tumors, R_i denotes the annual number of brain and CNS tumors cases. When estimating the risk of dying from brain and CNS tumors, R_i denotes the annual number of brain and CNS tumors deaths; N_i denotes the population; W_i denotes the width of the ith age group, the ith interval is from a_i to a_{i+1}, w_i=(a_{i+1}-a_i). $\hat{\lambda}_c$ denotes the observed brain and CNS tumors incidence rate, for age group i ($\hat{\lambda}_{c.i}$) can be calculated as:

$$\hat{\lambda}_{c.i}=\frac{R_i}{N_i}$$

$\hat{S}_0^*(a_i)$ denotes the probability of being alive and brain and CNS tumors free at age i. When calculating, died individuals should be deleted to calculate the number of surviving and brain and CNS tumors-free individuals. In addition, individuals diagnosed with brain and CNS tumors should also be deleted. To avoid double counting when estimation, we must subtract the number of brain and CNS tumors deaths (D_i) from all deaths (M_i), and then add the new number of brain and CNS tumors cases (R_i). So, the probability of being alive and brain and CNS tumors free at age i [$\hat{S}_0^*(a_i)$] can be approximately estimated as follows:

$$\hat{S}_0^*(a_i)=\text{exp}(\text{-}\sum_{j=1}^{i\text{-}1}\frac{R_j+(M_j\text{-}D_j)}{N_j})$$

The brain and CNS tumors incidence and mortality data are presented in the form of frequency table for calculation, and the final age band is usually ≥85 years old (e.g., 0−4, 5−9, 10−14, …, 80−84, ≥85). For the final age band (e.g., ≥85), the integral for the final age band evaluates to the following:

$$\frac{R_f}{R_f+M_f-D_f}S_0(a_f)$$

The derivation of variance is as follows:

Let

$$\hat{\gamma}_c=\frac{R_i}{R_i+M_i-D_i},\ \hat{S}_0^*=\hat{S}_0^*(a_i),\ S_x=\left(1-\exp\left(-\frac{W_i}{N_i}(R_i+M_i-D_i)\right)\right)$$

then

$$Var(S)=\sum_{i=1}^{f}Var\left(\hat{\gamma}_c\hat{S}_0^*S_x\right)=\sum_{i=1}^{f}\left[E\left(\hat{\gamma}_c^2\left(\hat{S}_0^*\right)^2S_x^2\right)-E\left(\hat{\gamma}_c\hat{S}_0^*S_x\right)^2\right] \quad (1)$$

$$E\left(\hat{\gamma}_c^2\left(\hat{S}_0^*\right)^2S_x^2\right)=E(\hat{\gamma}_c^2)E\left(\hat{S}_0^*\right)^2E(S_x^2)=\left([Var\left(\hat{S}_0^*\right)+(E\left(\hat{S}_0^*\right))^2][Var(S_x)+E(S_x)^2]\right)$$

$$Var(S)=\sum_{i=1}^{f}\{[Var(\hat{\gamma}_c)+(E\hat{\gamma}_c)^2][Var\left(\hat{S}_0^*\right)+\left(E\hat{S}_0^*\right)^2][Var(S_x)+(ES_x)^2-(E\hat{\gamma}_c)^2\left(E\hat{S}_0^*\right)^2(ES_x)^2]\}$$

Let

$$p_{ci}=\frac{R_i}{R_i+M_i-D_i}$$

then

$$E\hat{\gamma}_c=p_{ci} \quad (2)$$

$$Var(\hat{\gamma}_c)=\frac{p_{ci}}{R_i+M_i-D_i} \quad (3)$$

$$\log\left(\hat{S}_0^*\right)=-\sum_{j=1}^{i-1}\frac{R_j+M_j-D_j}{N_j},$$

Let

$$p_{oj}=\frac{R_j+M_j-D_j}{N_j}$$

then

$$Var(\log\hat{S}_0^*)=\sum_{j=1}^{i-1}Var\left(\frac{R_j+M_j-D_j}{N_j}\right)=\sum_{j=1}^{i-1}\frac{p_{oj}}{N_j},$$

According to delta method, if $\hat{\theta}\sim N(\theta,\sigma^2)$, then $f(\hat{\theta})\sim N(f(\theta),[f'(\theta)]^2\sigma^2)$. Let $\hat{S}_0^*=e^{\hat{\theta}}$, then

$$E\left(\hat{S}_0^*\right)=\exp\left(-\sum_{j=1}^{i-1}p_{oj}\right) \quad (4)$$

$$Var\left(\hat{S}_0^*\right)=(e^{\hat{\theta}})^2 Var\left(\log\hat{S}_0^*\right)=\left(\hat{S}_0^*\right)^2 Var\left(\log\hat{S}_0^*\right)=\left(\hat{S}_0^*\right)^2\times\sum_{j=1}^{i-1}\frac{p_{oj}}{N_j} \quad (5)$$

$$S_x=\left(1-\exp\left(-\frac{w_i}{N_i}(R_i+M_i-D_i)\right)\right),$$

Let $S_x^{'}=\exp\left(-\frac{w_i}{N_i}(R_i+M_i-D_i)\right)$, $\log\left(S_x^{'}\right)=-\frac{w_i}{N_i}(R_i+M_i-D_i)$, and $p_{xi}=\frac{R_i}{R_i+M_i-D_i}$, then

$$Var(\log S_x^{'})=w_i^2\frac{p_{xi}}{N_i}$$

Let $S_x=1-e^{\hat{\theta}}$, according to delta method, then:

$$E(S_x)=\left(1-\exp\left(-\frac{w_i}{N_i}(R_i+M_i-D_i)\right)\right) \quad (6)$$

$$Var(S_x)=(e^{\hat{\theta}})^2\times Var\left(e^{\hat{\theta}}\right)=(S_x^{'})^2\times w_i^2\frac{p_{xi}}{N_i} \quad (7)$$

References

1. Sasieni PD, Shelton J, Ormiston-Smith N, Thomson CS, Silcocks PB. What is the lifetime risk of developing cancer?: the effect of adjusting for multiple primaries. Br J Cancer. 2011;105(3):460-5. https://doi.org/10.1038/bjc.2011.250.

2. Ahmad AS, Ormiston-Smith N, Sasieni PD. Trends in the lifetime risk of developing cancer in Great Britain: comparison of risk for those born from 1930 to 1960. Br J Cancer. 2015;112(5):943-7. https://doi.org/10.1038/bjc.2014.606.

3. Zheng R, Wang S, Zhang S, Zeng H, Chen R, Sun K, *et al*. Global, regional, and national lifetime probabilities of developing cancer in 2020. Sci Bull (Beijing). 2023;68(21):2620-8. https://doi.org/10.1016/j.scib.2023.09.041.

Figure legends

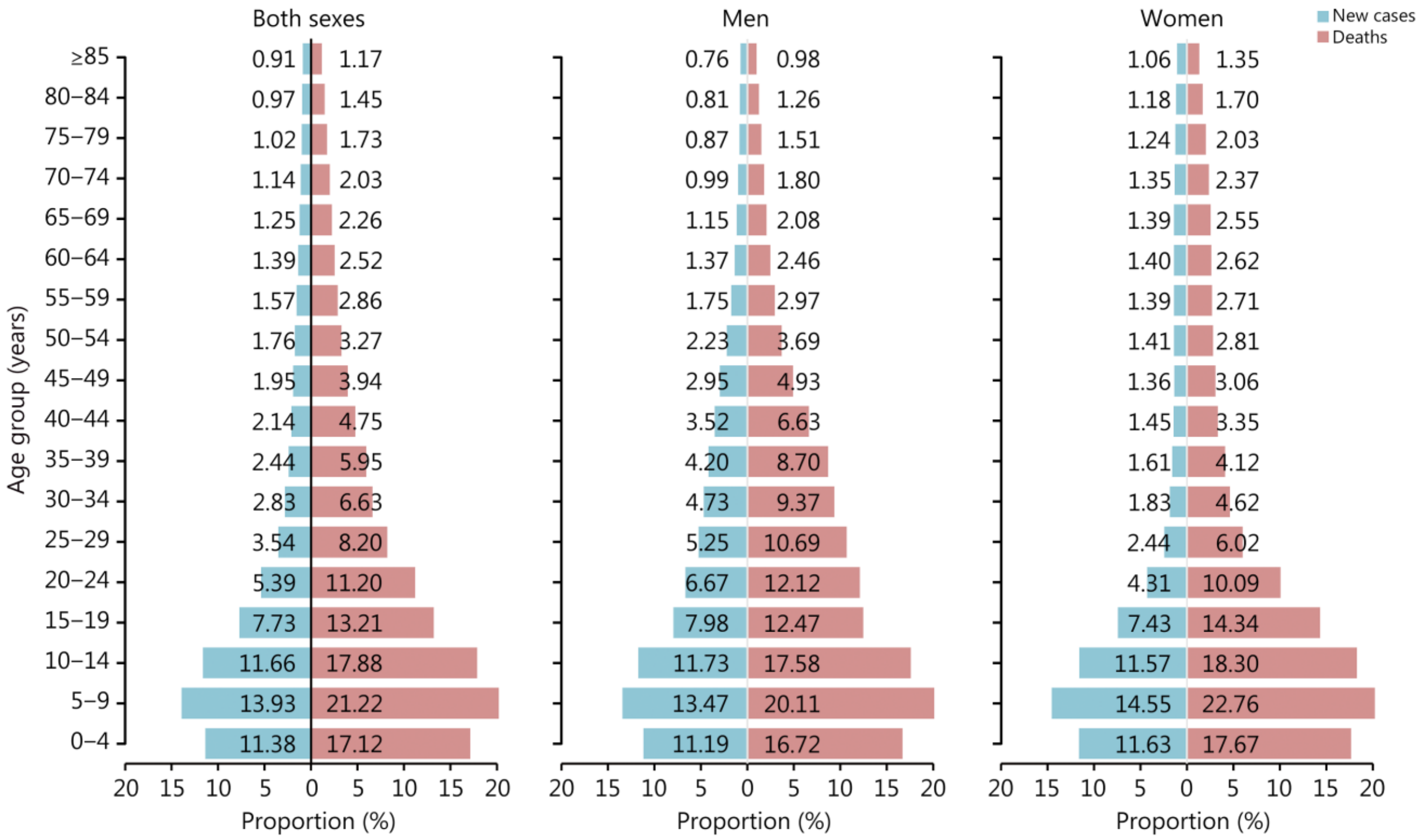

Fig. S1 Proportion of new cases or deaths of brain and CNS tumors by age group and sex, 2024. CNS. Central nervous system

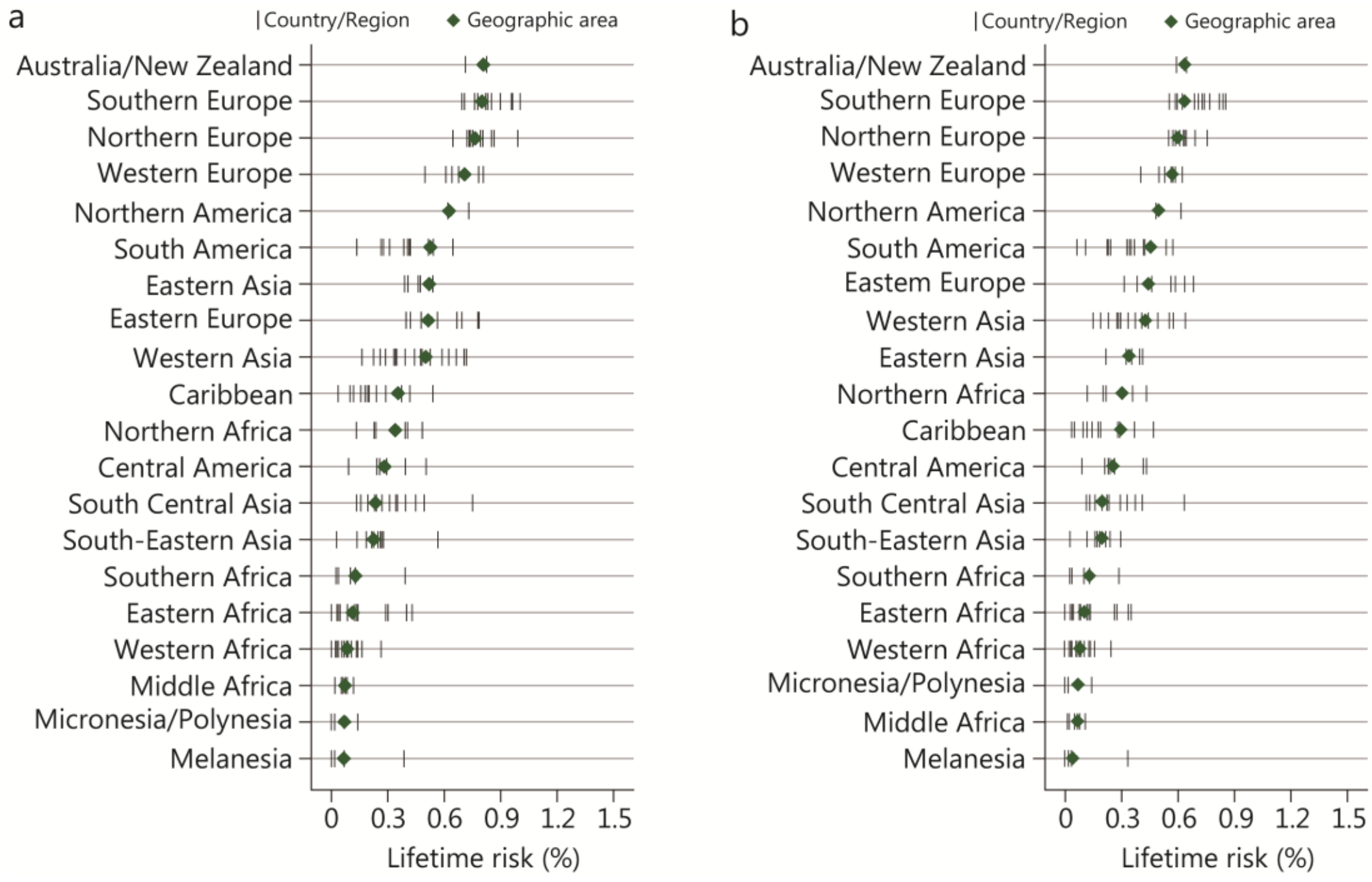

Fig. S2 Lifetime risks of developing or dying from brain and CNS tumors in 2024 by geographic area for both sexes. **a** Developing brain and CNS tumors. **b** Dying from brain and CNS tumors. Green diamonds denote the average lifetime risk in each selected geographic areas; vertical lines denote the estimated lifetime risk in each selected countries/regions. CNS. Central nervous system

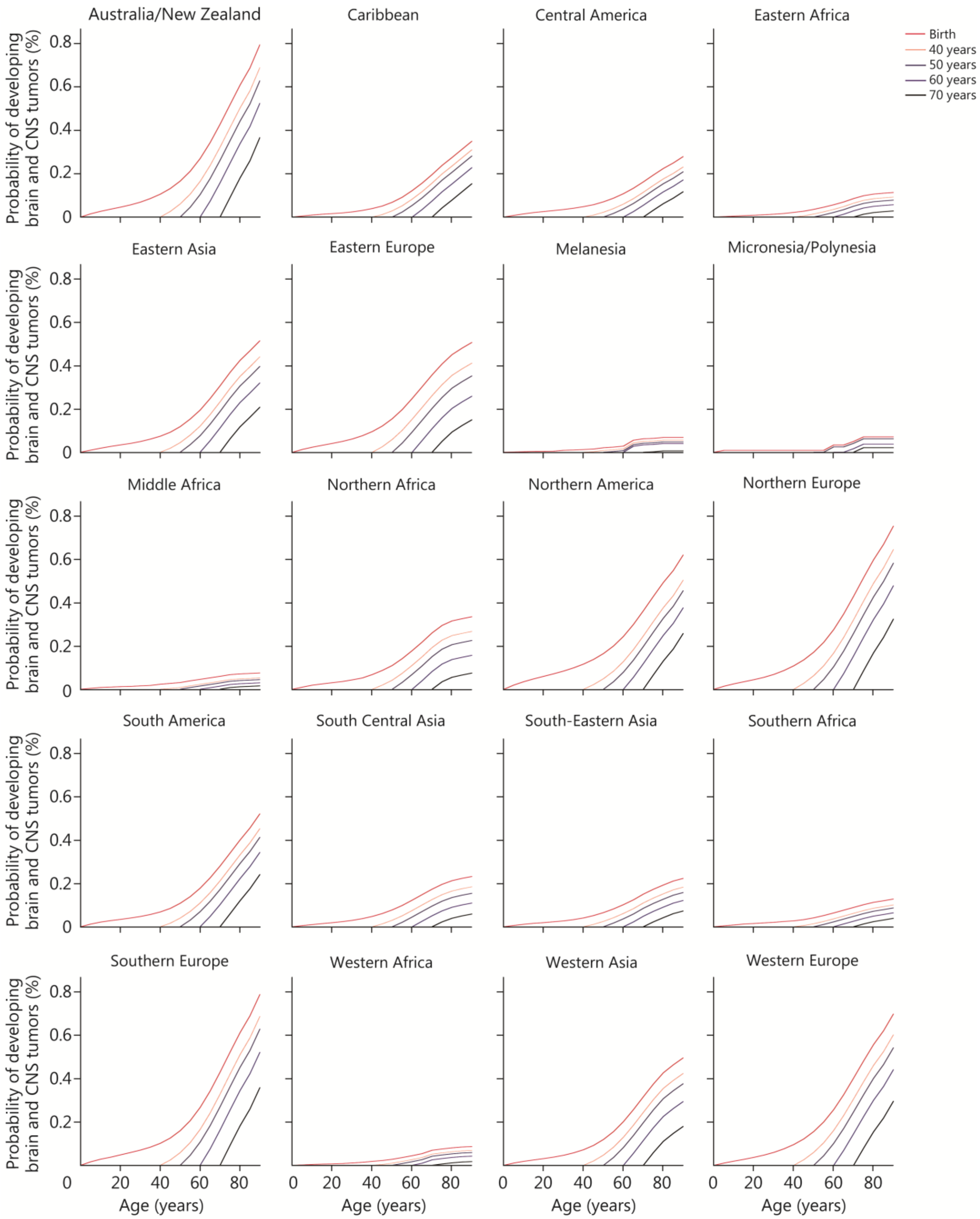

Fig. S3 Lifetime and age-conditional probability of developing brain and CNS tumors in 20 world regions for both sexes, 2024. CNS. Central nervous system

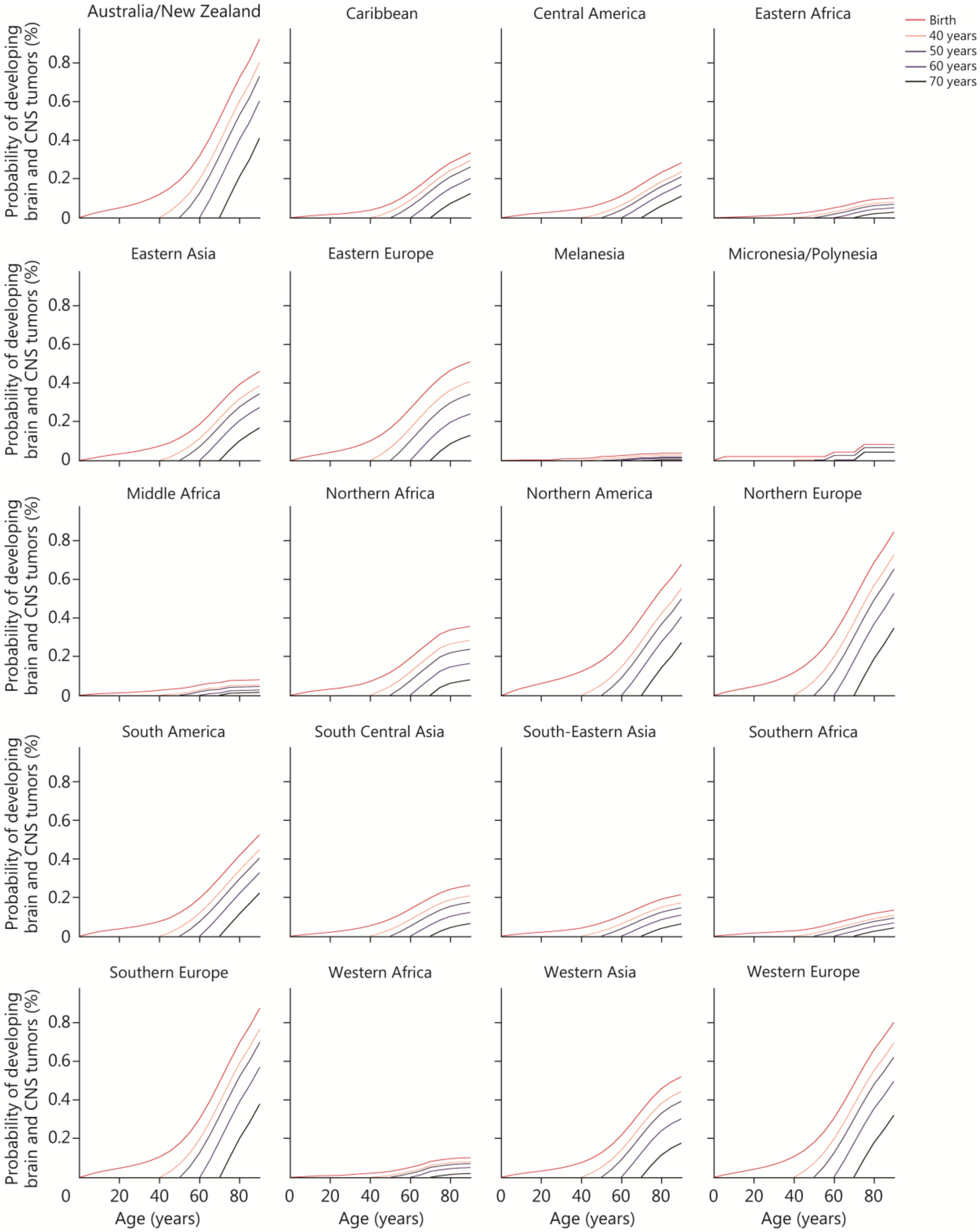

Fig. S4 Lifetime and age-conditional probability of developing brain and CNS tumors in 20 world regions for men, 2024. CNS. Central nervous system

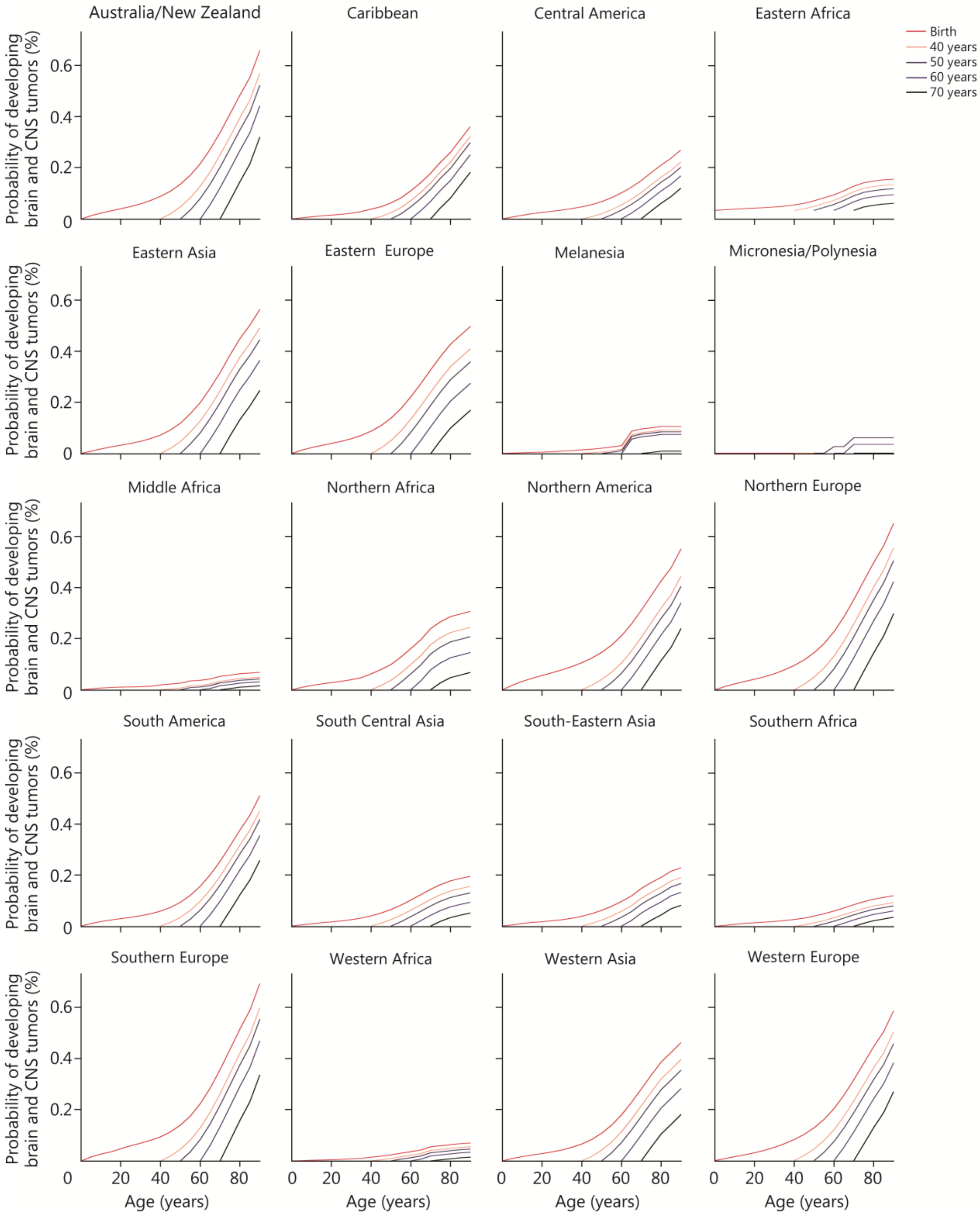

Fig. S5 Lifetime and age-conditional probability of developing brain and CNS tumors in 20 world regions for women, 2024. CNS. Central nervous system

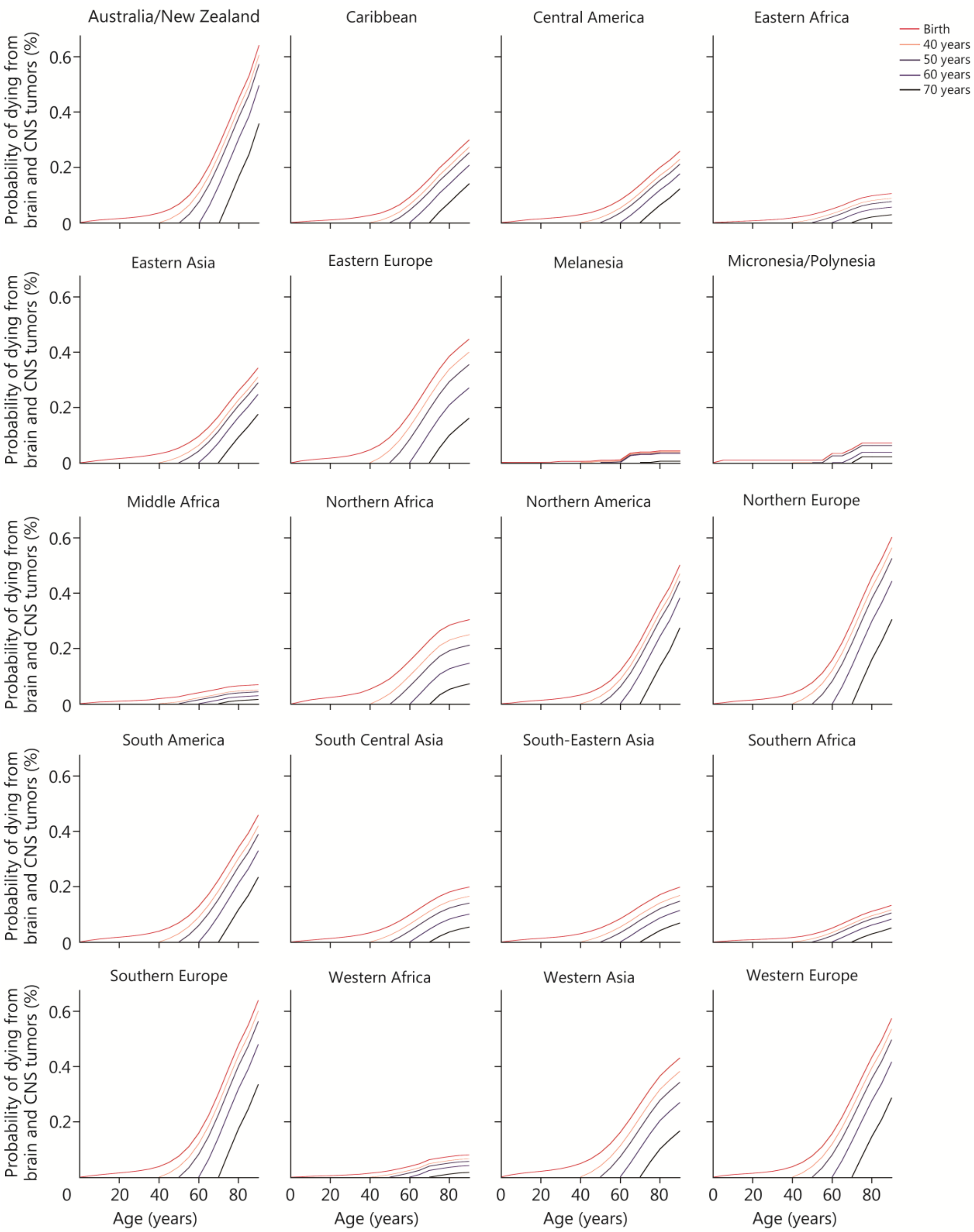

Fig. S6 Lifetime and age-conditional probability of dying from brain and CNS tumors in 20 world regions for both sexes, 2024. CNS. Central nervous system

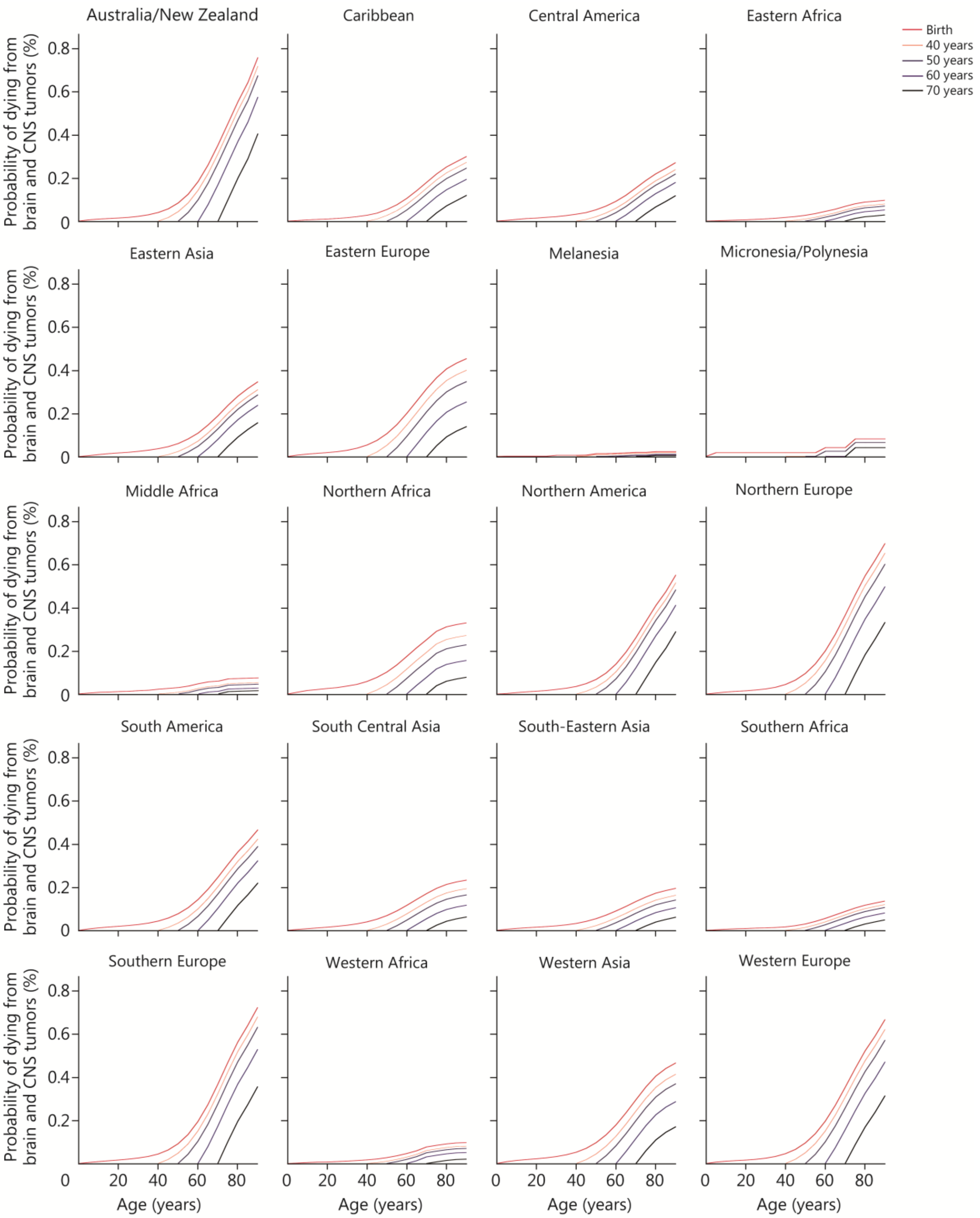

Fig. S7 Lifetime and age-conditional probability of dying from brain and CNS tumors in 20 world regions for men, 2024.

CNS. Central nervous system

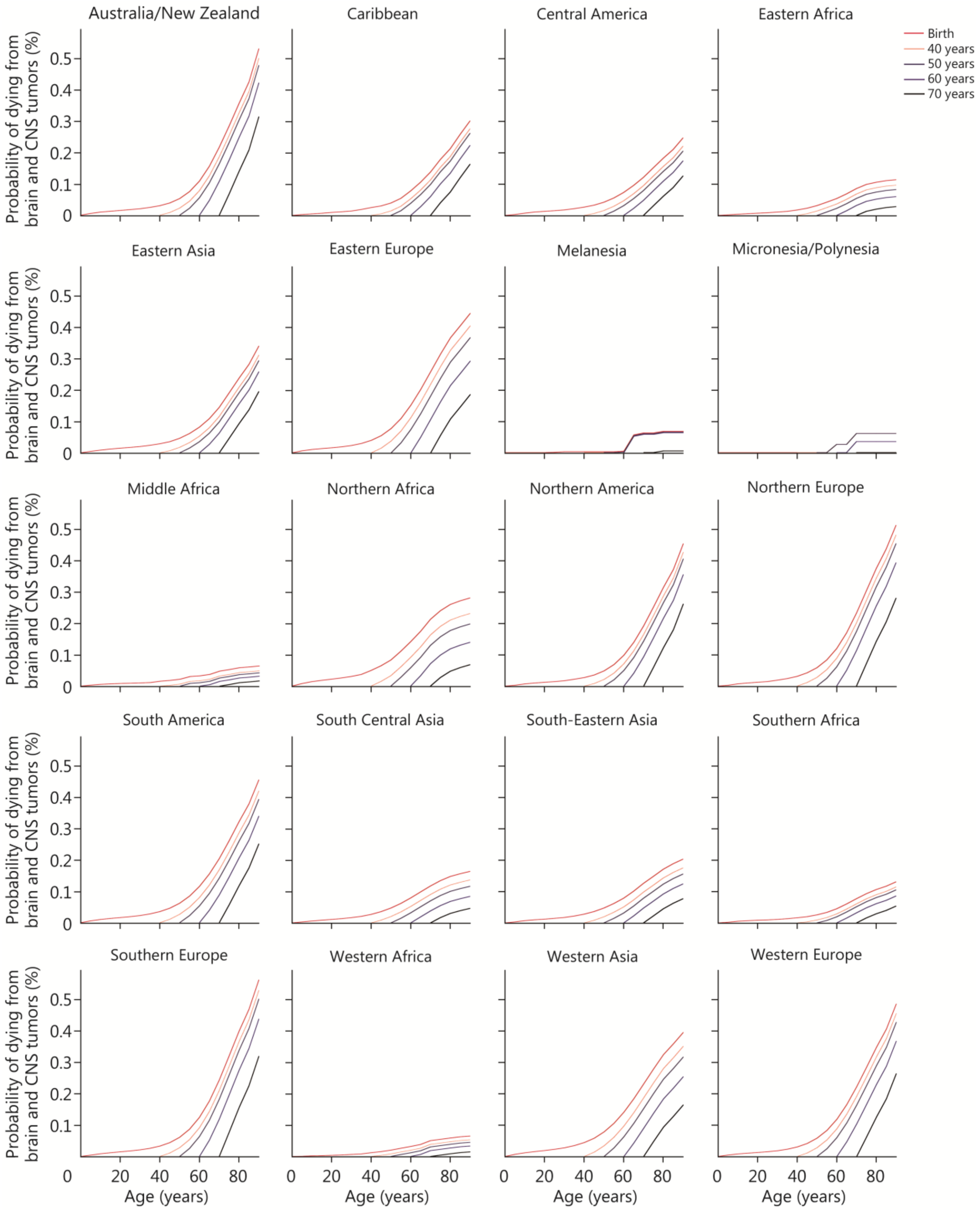

Fig. S8 Lifetime and age-conditional probability of dying from brain and CNS tumors in 20 world regions for women, 2024. CNS. Central nervous system

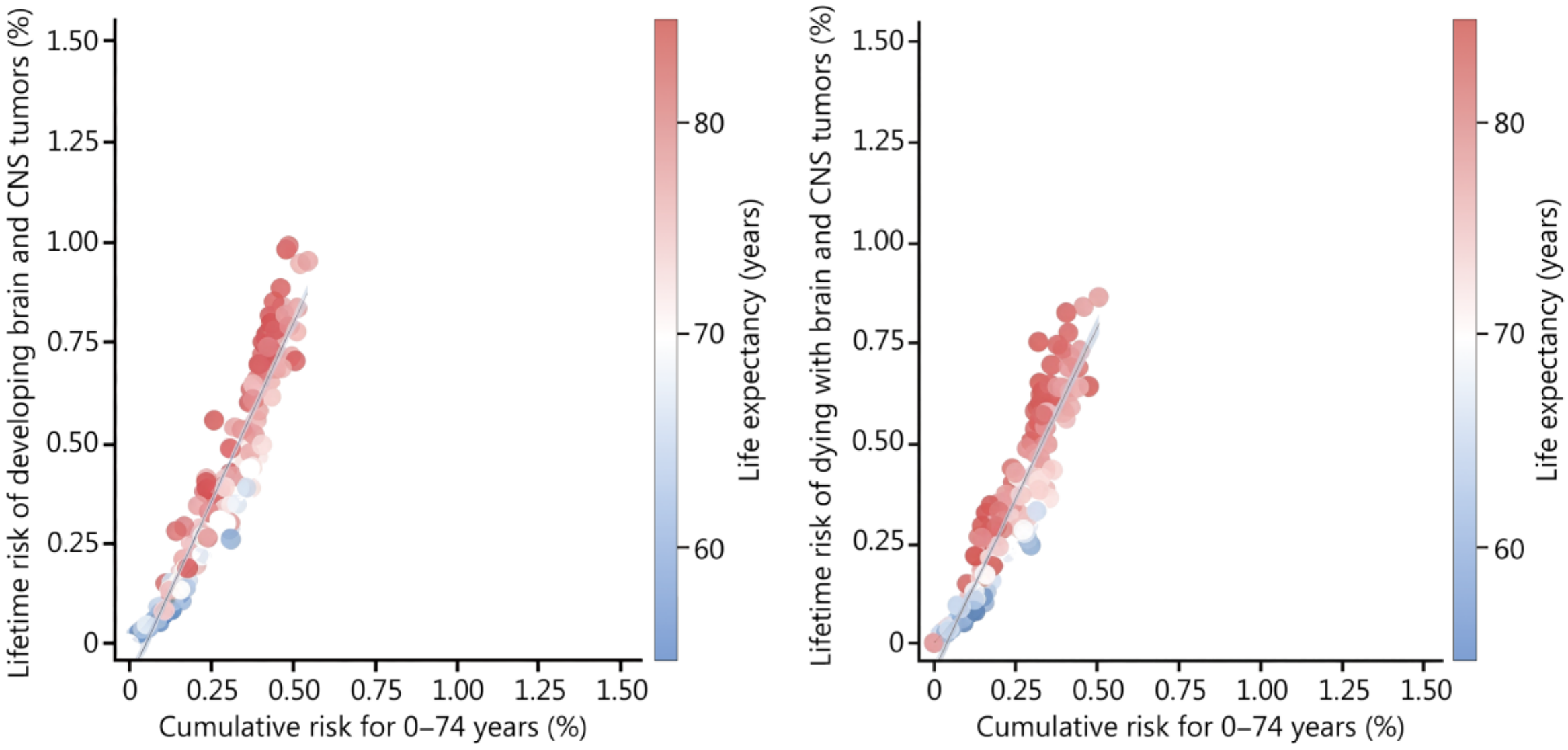

Fig. S9 Correlation between lifetime risk of developing or dying from brain and CNS tumors and traditional cumulative risk measure (aged 0–74 years) for both sexes. The red line indicates the fitted risks using reg regression with a 95% confidence interval; the colored circles indicate the risks in different countries/regions with different life expectancy. CNS. Central nervous system

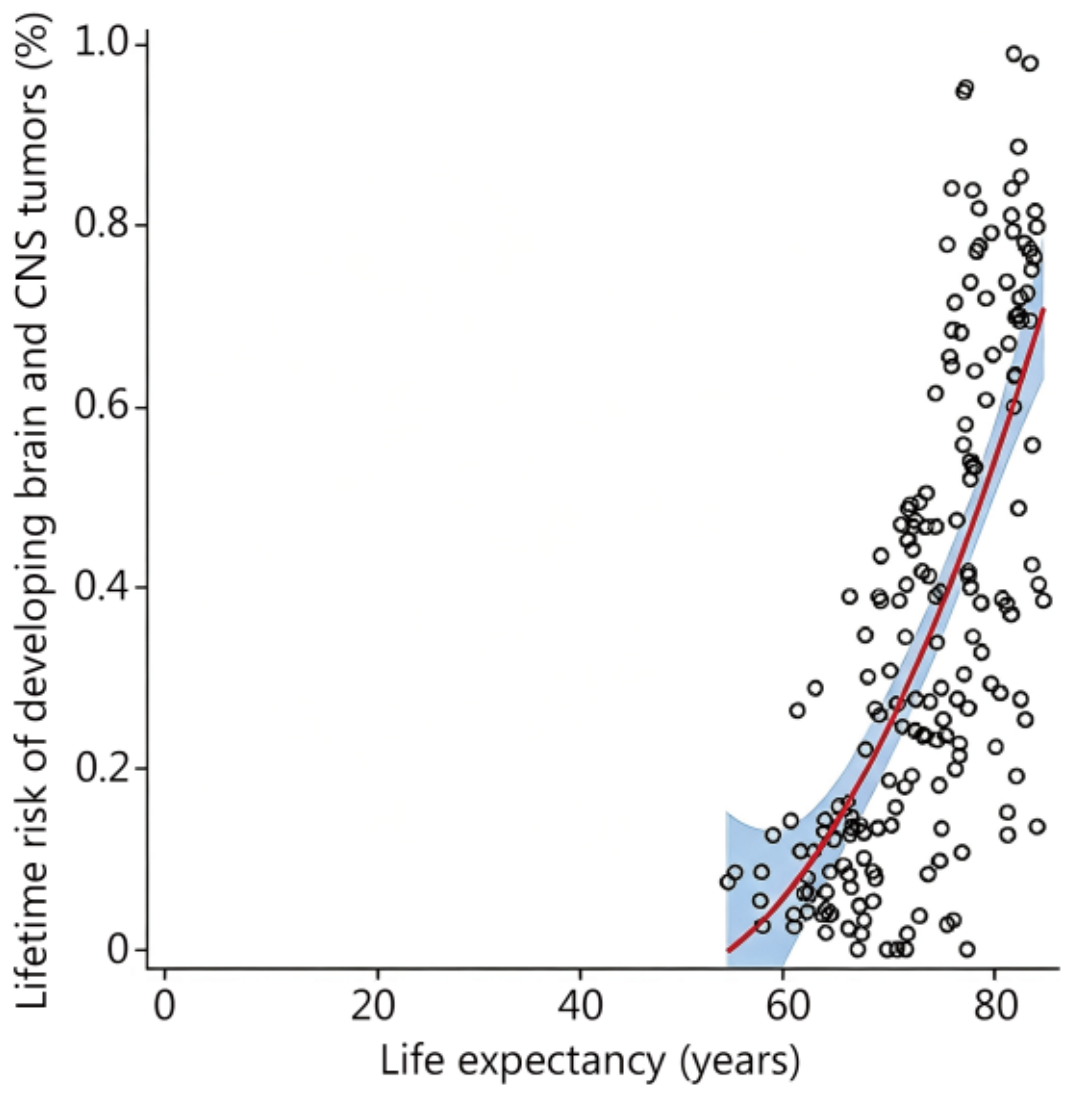

Fig. S10 Correlation between lifetime risk of developing brain and CNS tumors and life expectancy for both sexes. Red line was fitted with quadratic regression and 95% confidence interval. CNS. Central nervous system

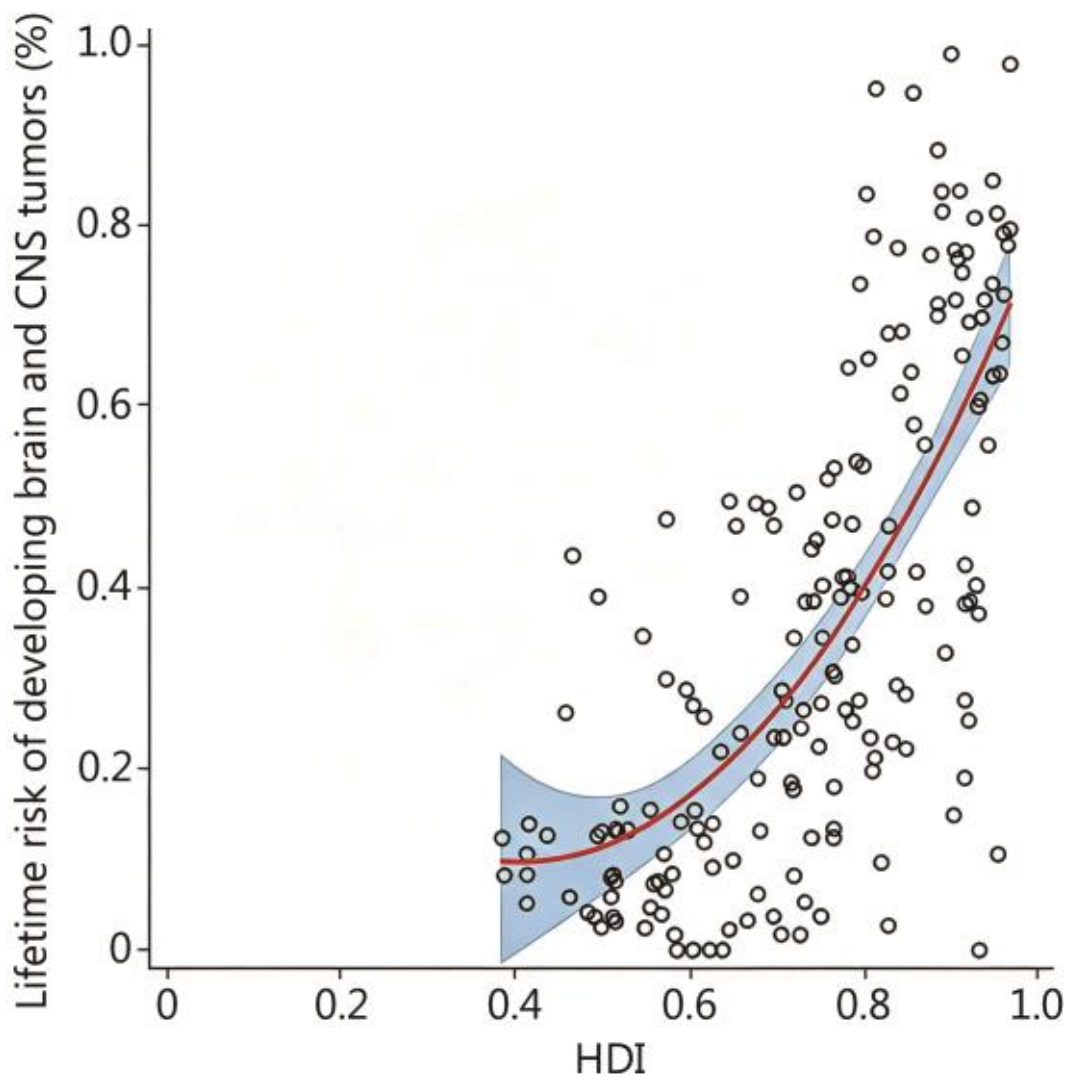

Fig. S11 Correlation between lifetime risk of developing brain and CNS tumors and human development index (HDI) for both sexes. Red line was fitted with quadratic regression and 95% confidence interval. CNS. Central nervous system

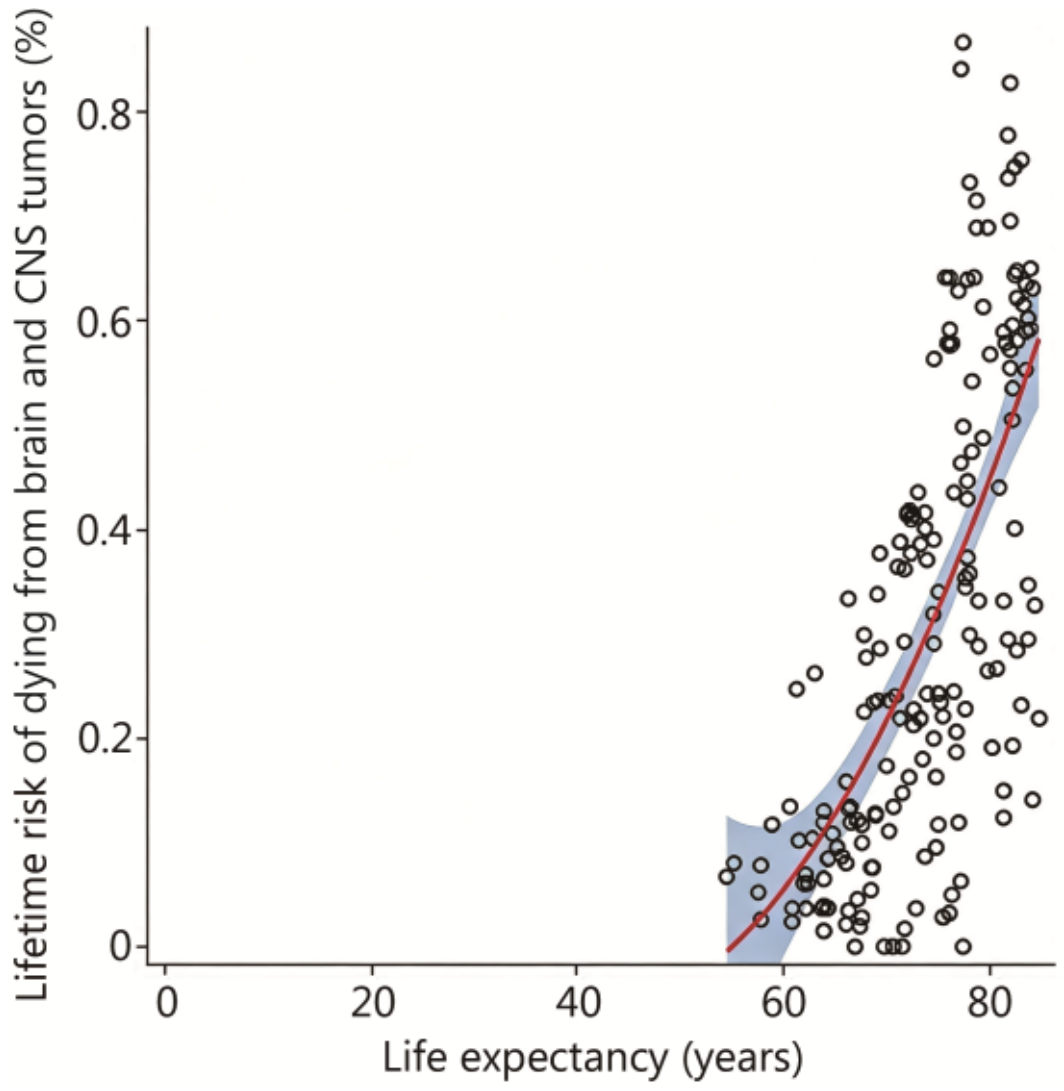

Fig. S12 Correlation between lifetime risk of dying from brain and CNS tumors and life expectancy for both sexes. The red line was fitted with quadratic regression and 95% confidence interval. CNS. Central nervous system

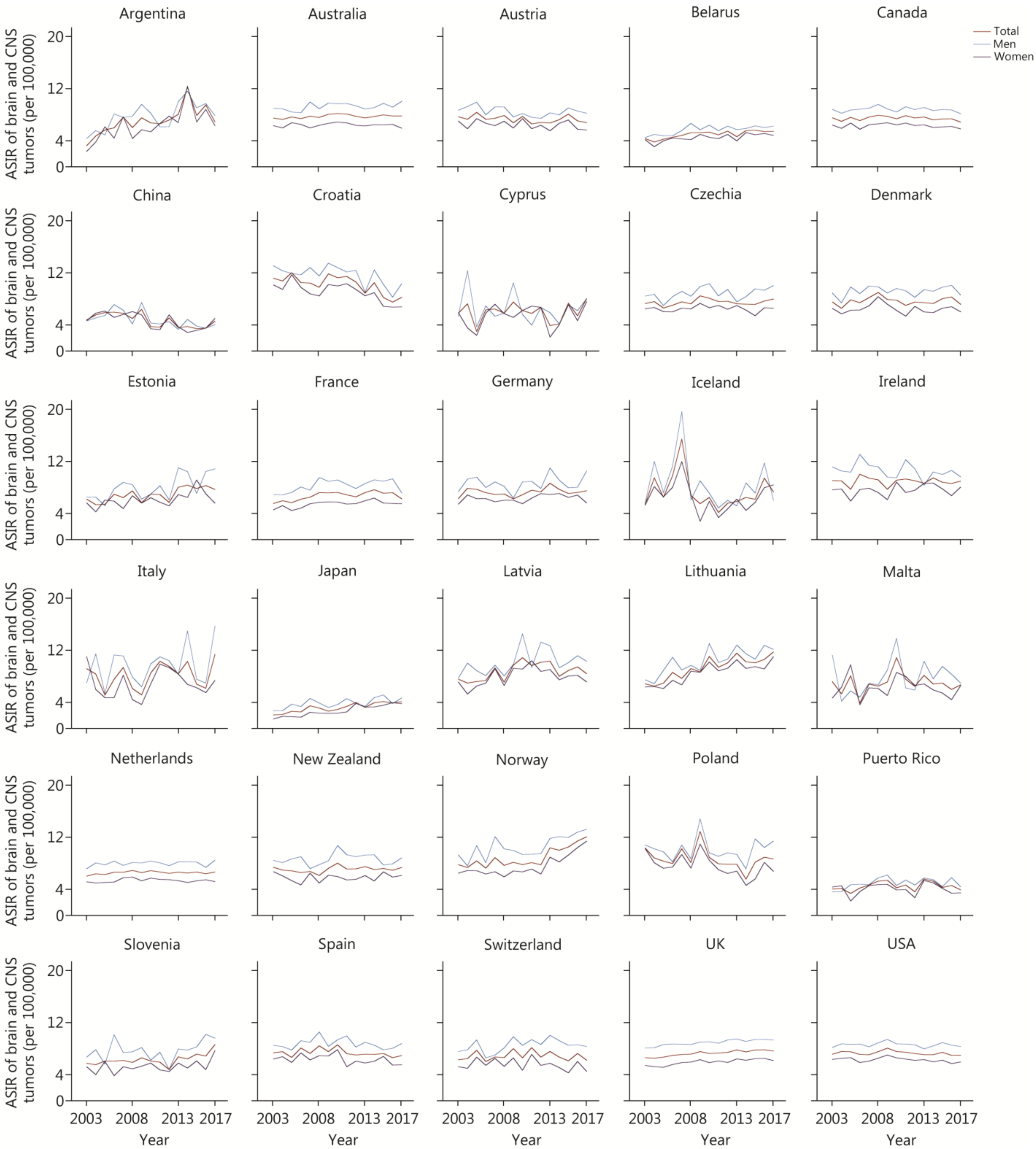

Fig. S13 The trends of age standardized incidence rate (ASIR) of brain and CNS tumors, 2003–2017. CNS. Central nervous system

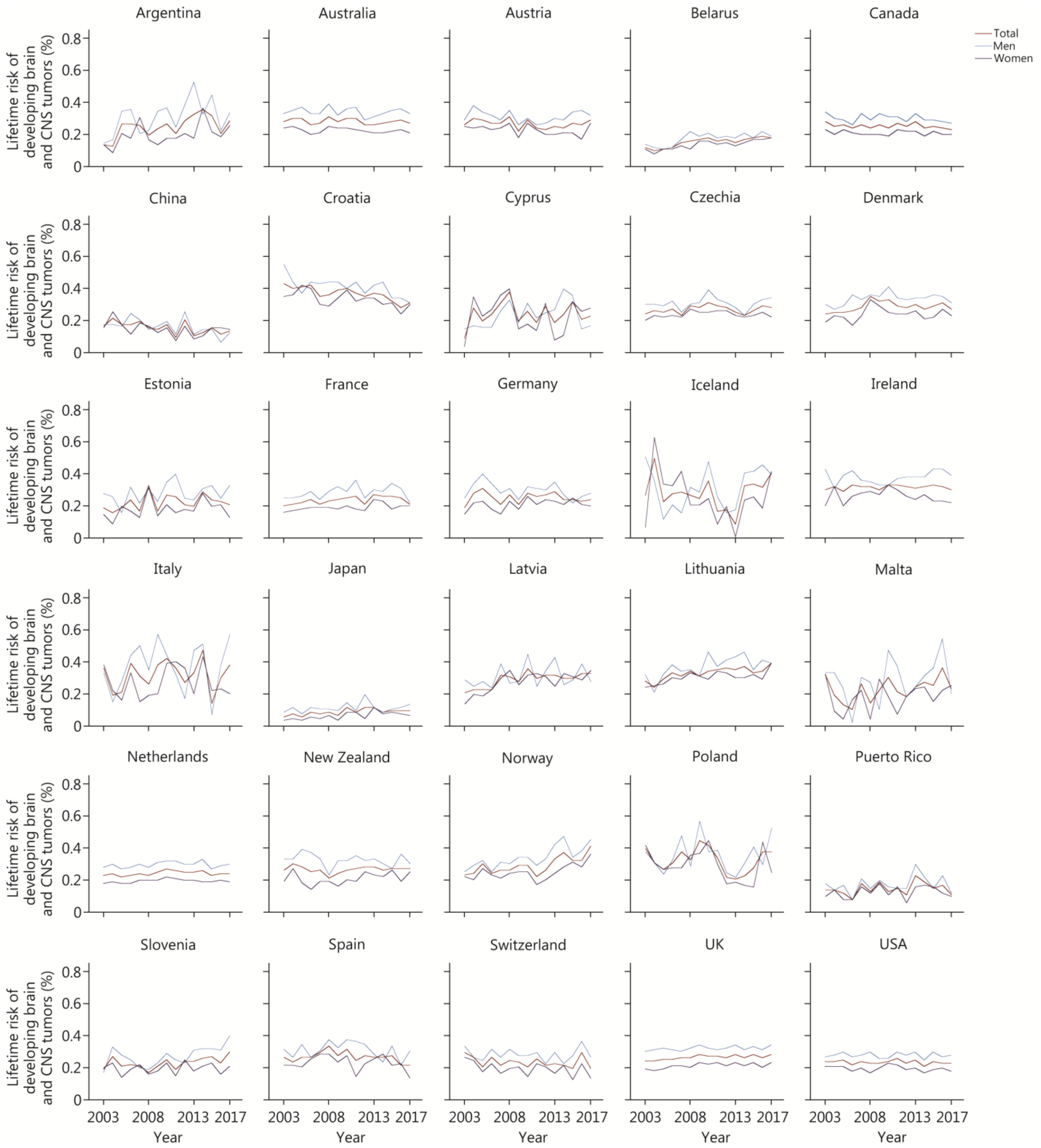

Fig. S14 The trends of lifetime risk of developing brain and CNS tumors, 2003–2017. CNS. Central nervous system

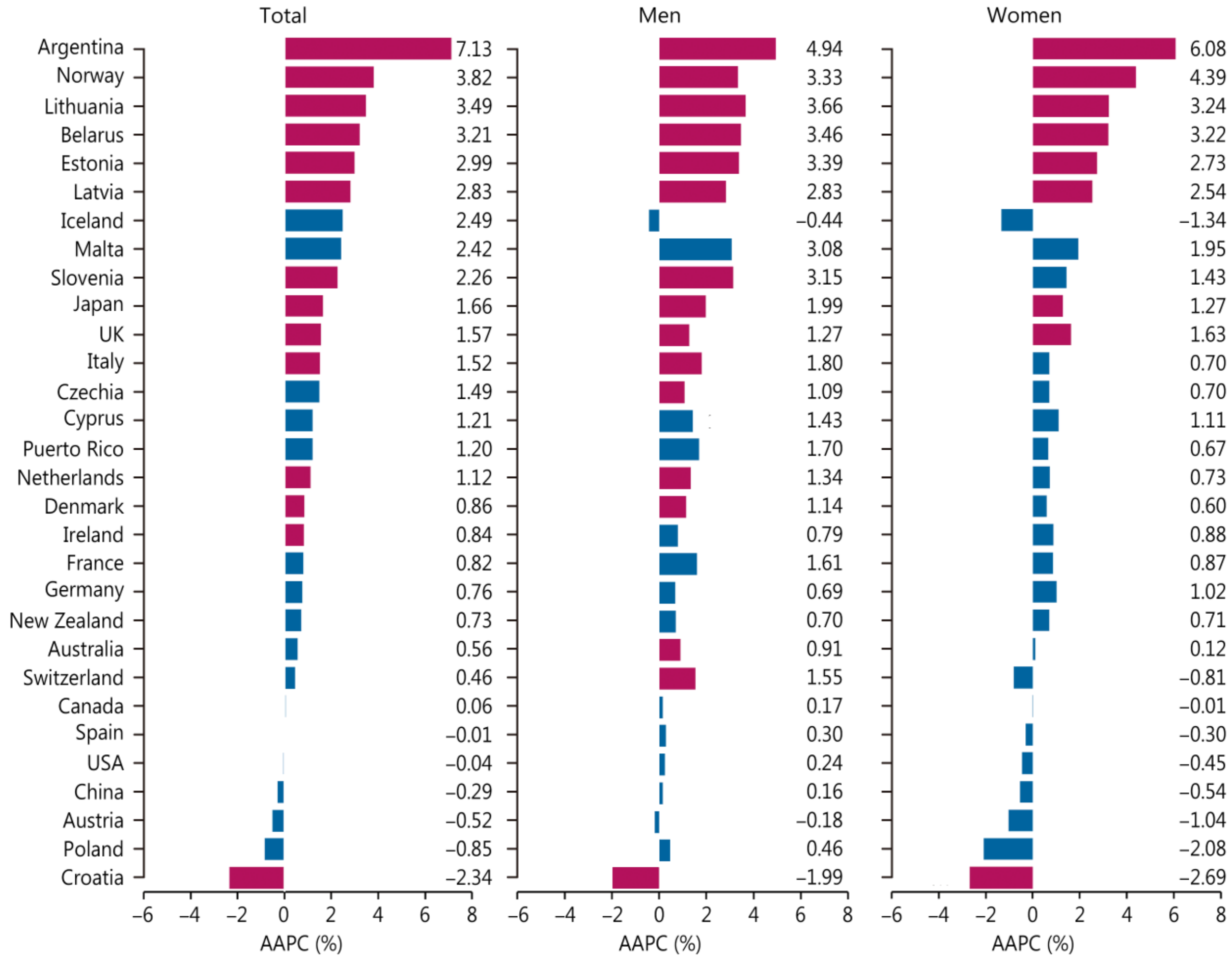

Fig. S15 The average annual percent change (AAPC) in lifetime risk of developing brain and CNS tumors by country, 2003–2017. Red bars represent AAPC with a statistically significant trend (*P*<0.05). Navy-blue bars represent AAPC is not statistically significant trend (*P*≥0.05). CNS. Central nervous system

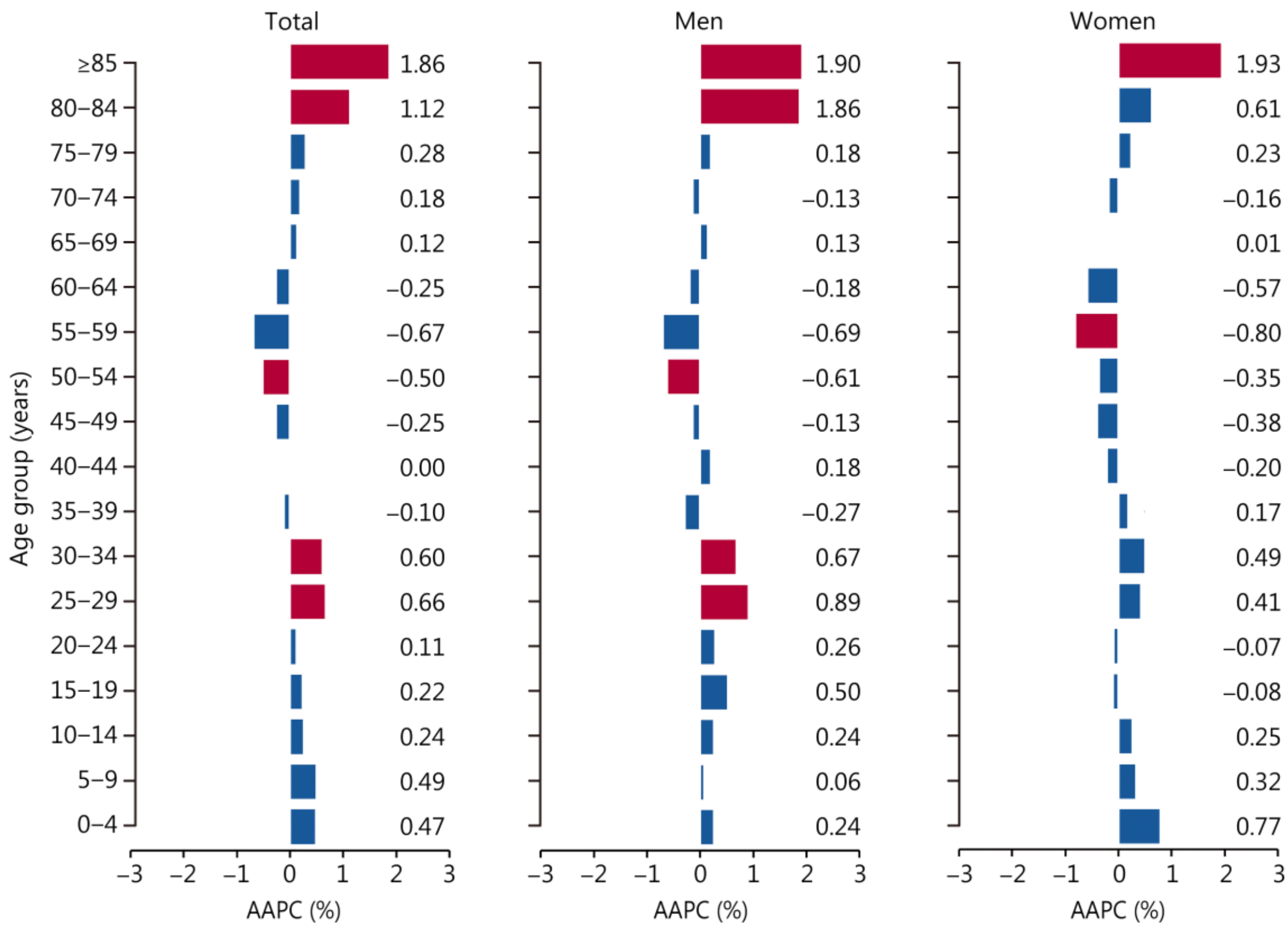

Fig. S16 The average annual percent change (AAPC) in age-specific incidence of brain and CNS tumors, 2003–2017. Red bars represent AAPC with a statistically significant trend ($P<0.05$). Navy-blue bars represent AAPC is not statistically significant trend ($P\geq0.05$). CNS. Central nervous system

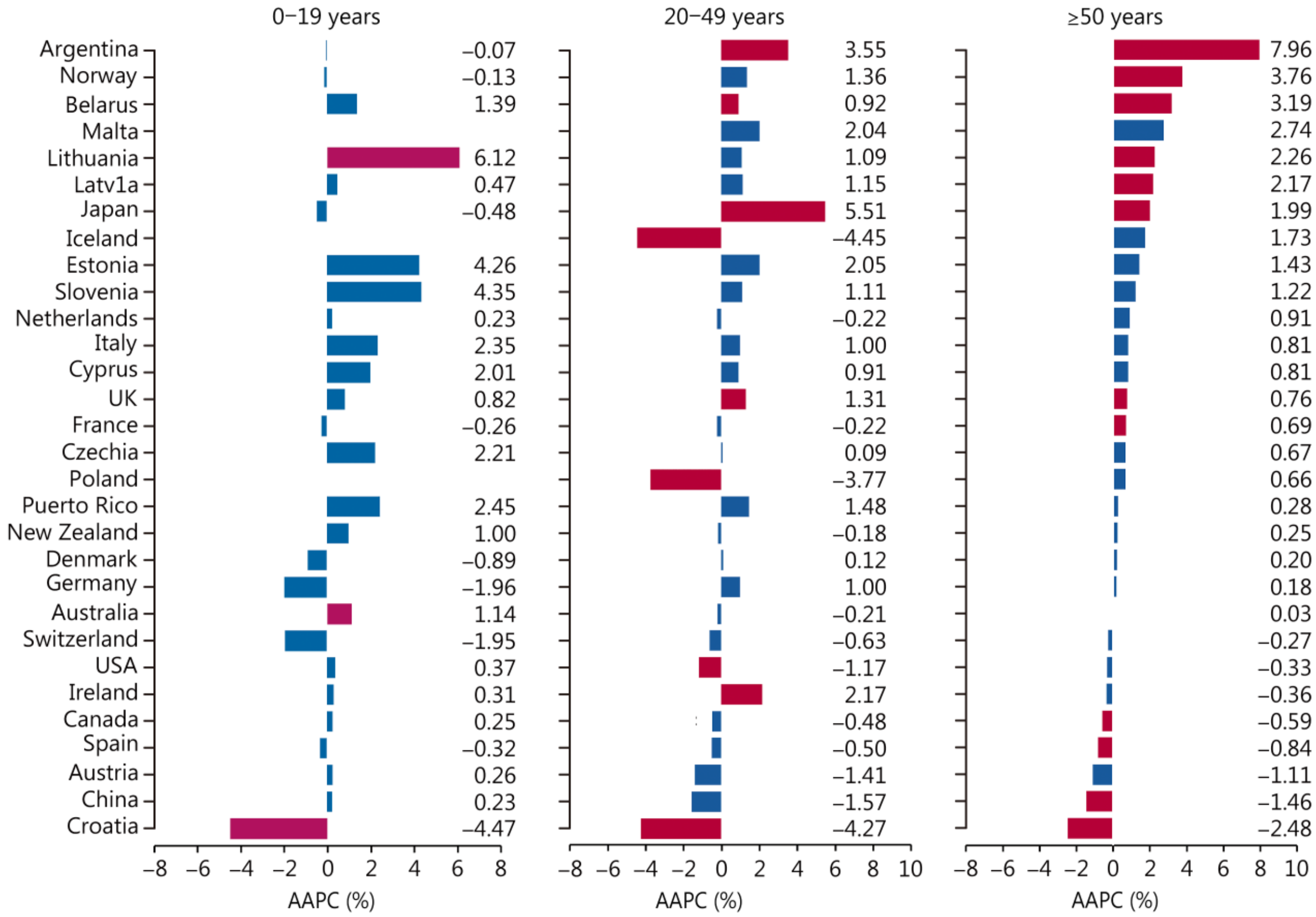

Fig. S17 The average annual percent change (AAPC) in age-standardized incidence rates of brain and CNS tumors by age group for both sexes, 2003–2017. Red bars represent AAPC with a statistically significant trend (P<0.05). Navy-blue bars represent AAPC is not statistically significant trend (P≥0.05). Data in Iceland, Malta, and Poland were not available for trend analysis in age 0–19 years. CNS. Central nervous system

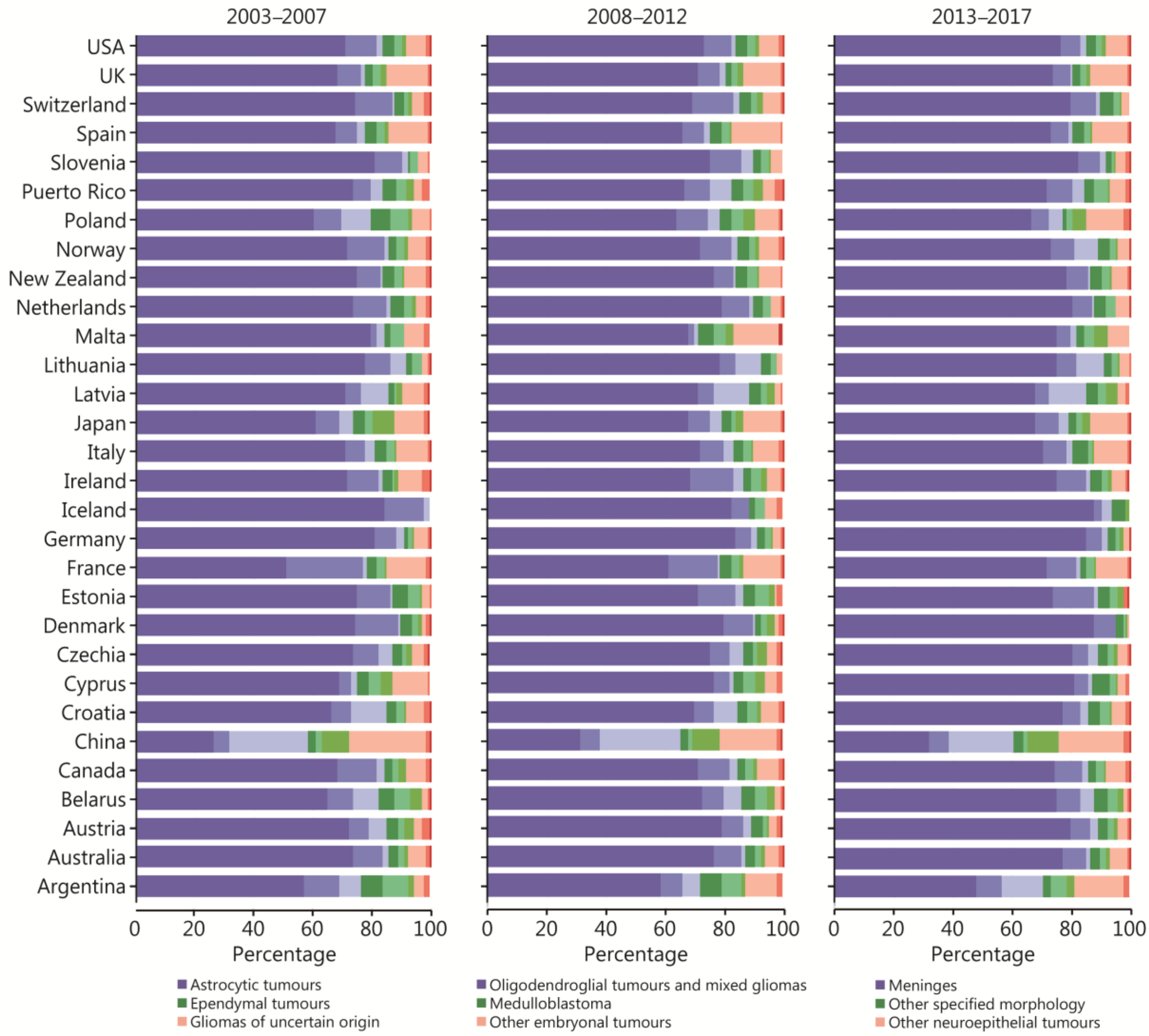

Fig. S18 The percentage of brain and CNS tumor cases by subtype in men, 2003–2017. CNS. Central nervous system

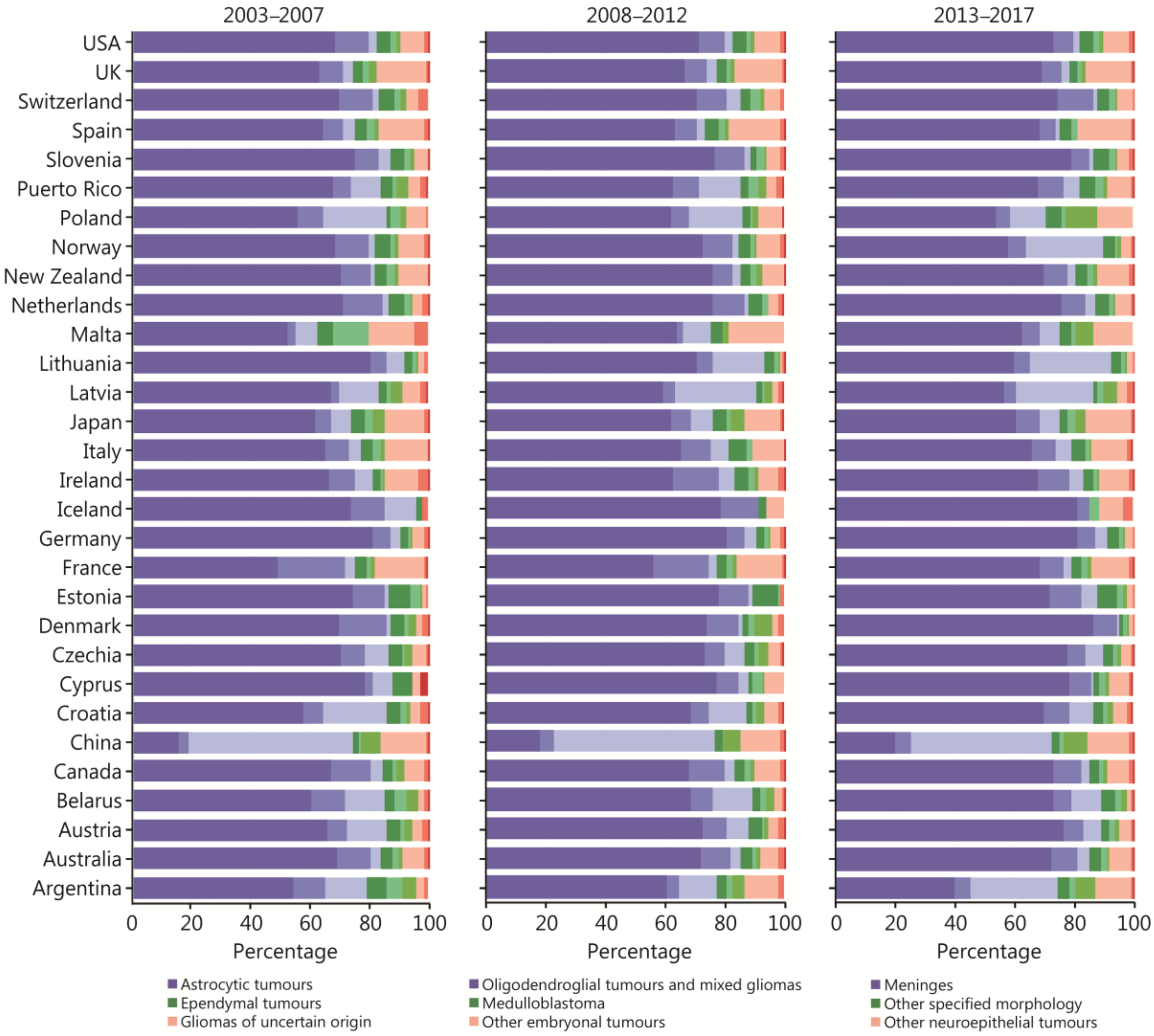

Fig. S19 The percentage of brain and CNS tumor cases by subtype in women, 2003–2017. CNS. Central nervous system

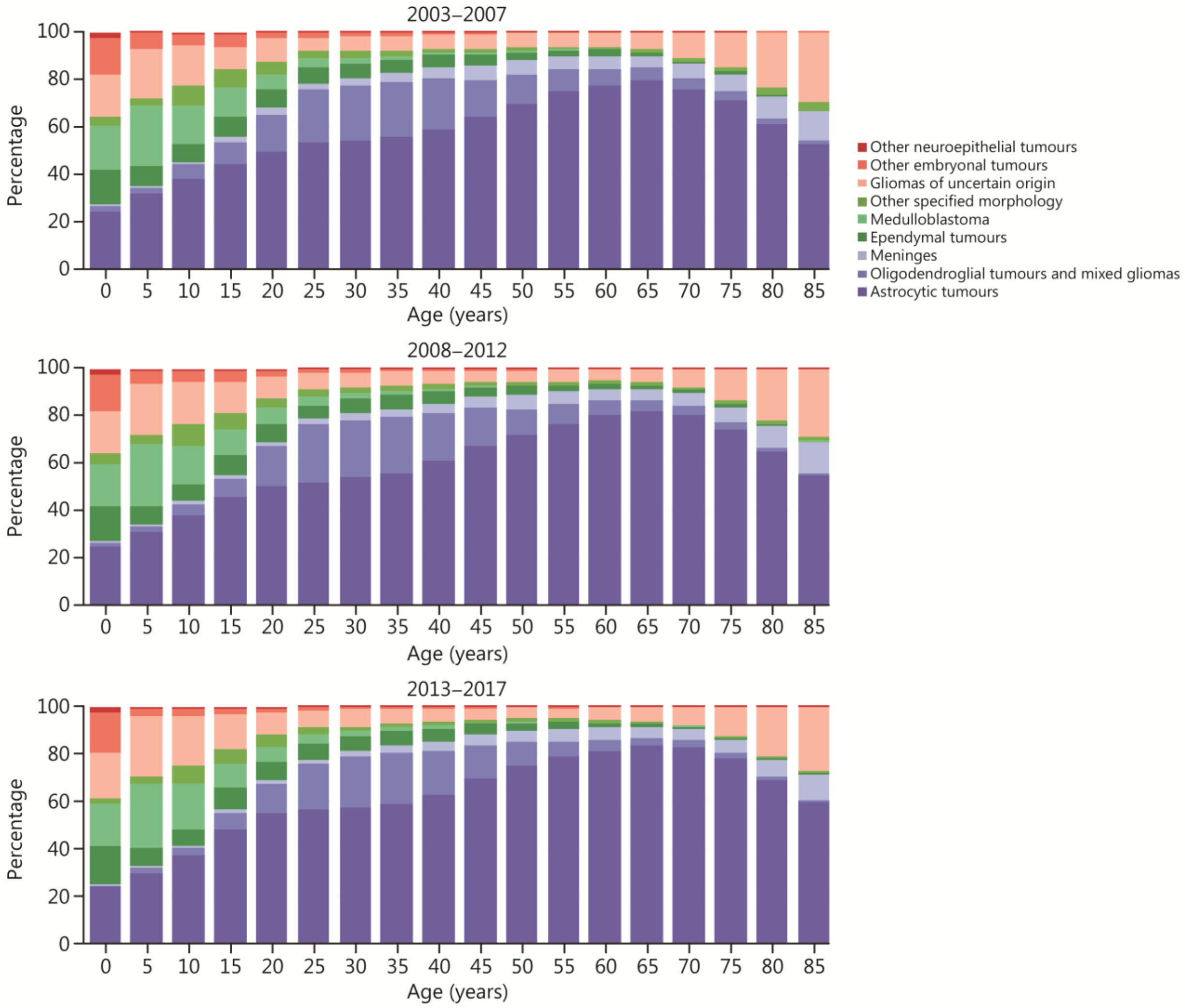

Fig. S20 The age-specific percentage of brain and CNS tumor cases by subtype in both sexes, 2003–2017. CNS. Central nervous system

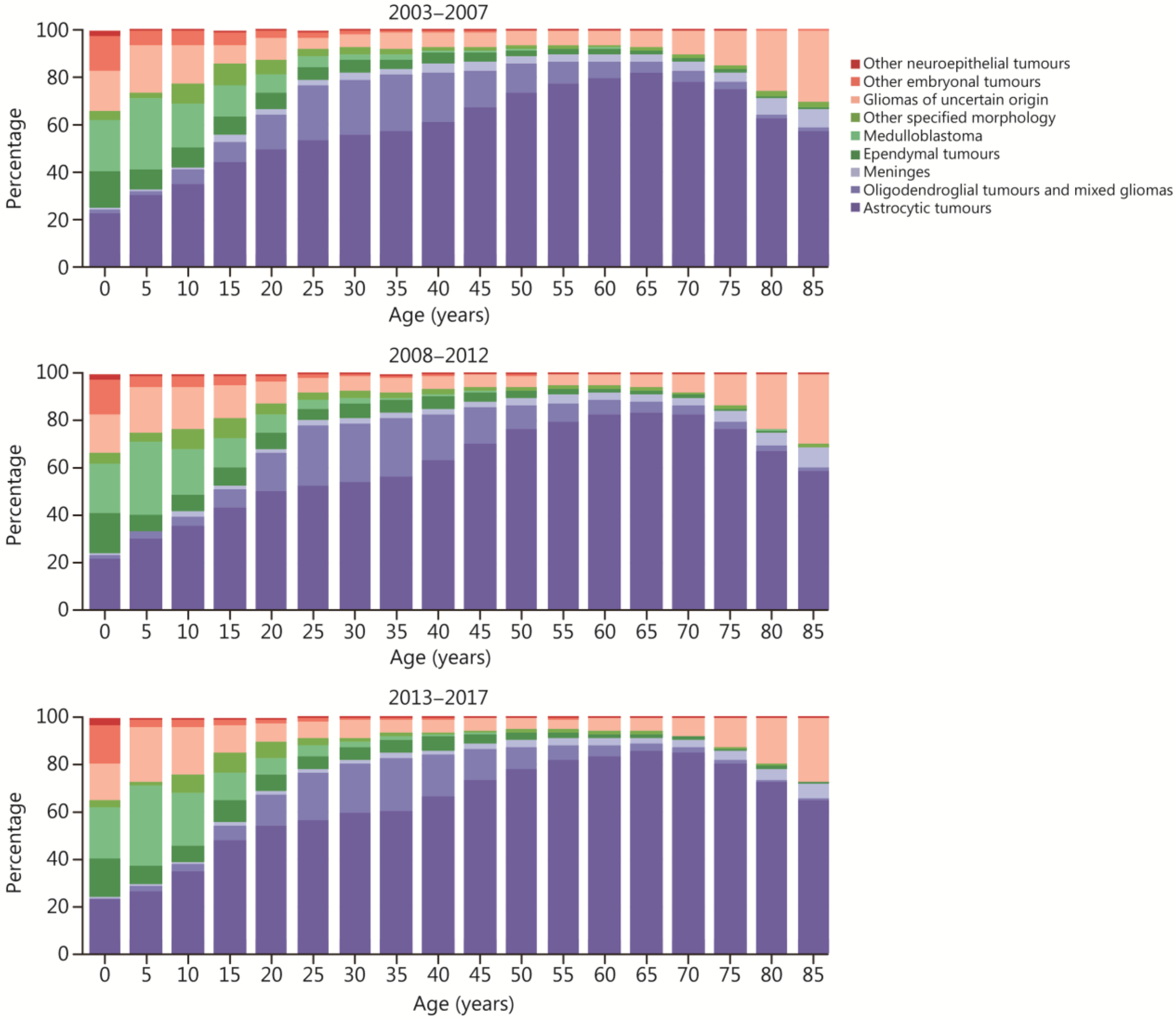

Fig. S21 The age-specific percentage of brain and CNS tumor cases by subtype in men, 2003–2017. CNS. Central nervous system

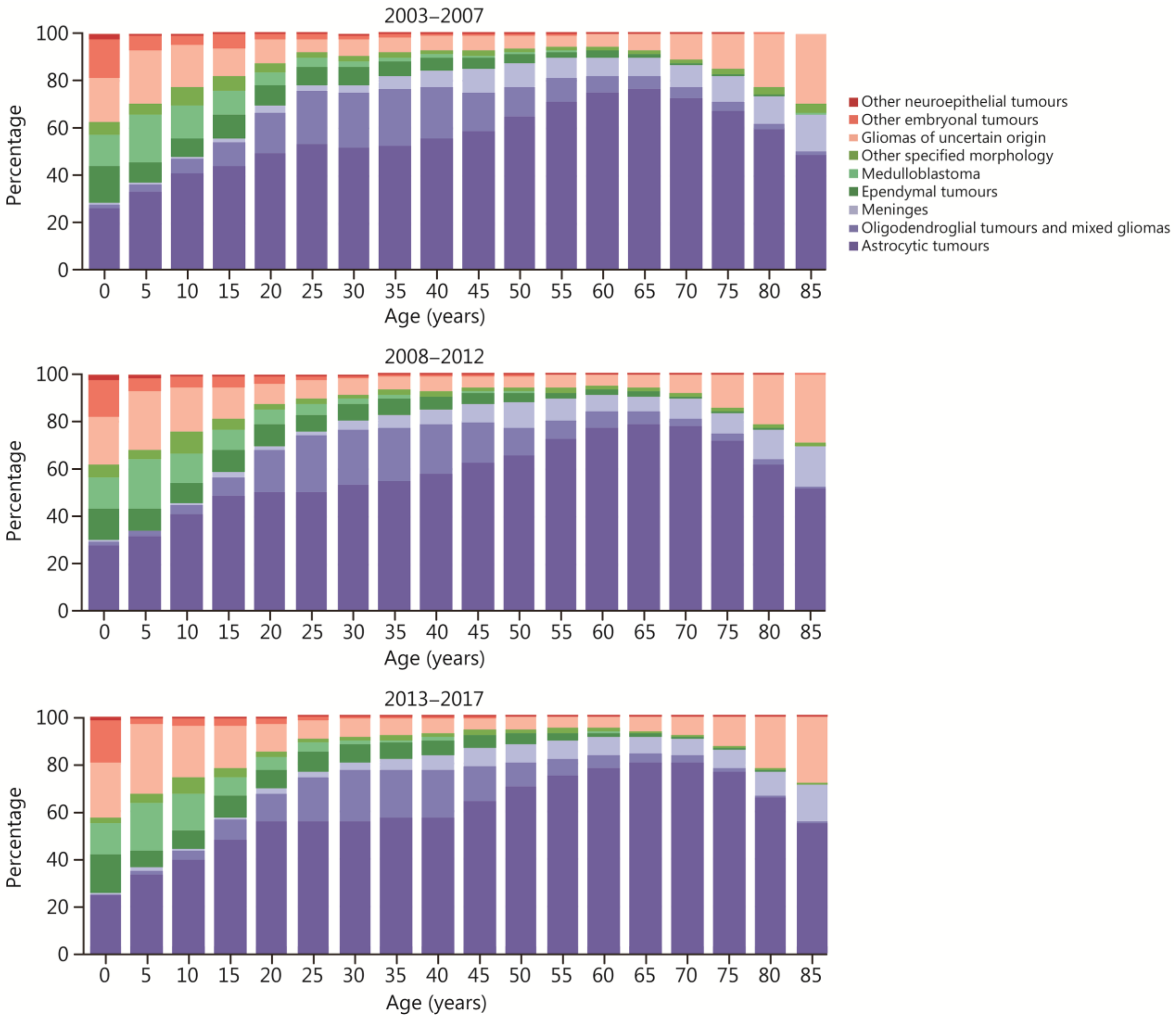

Fig. S22 The age-specific percentage of brain and CNS tumor cases by subtype in women, 2003–2017. CNS. Central nervous system

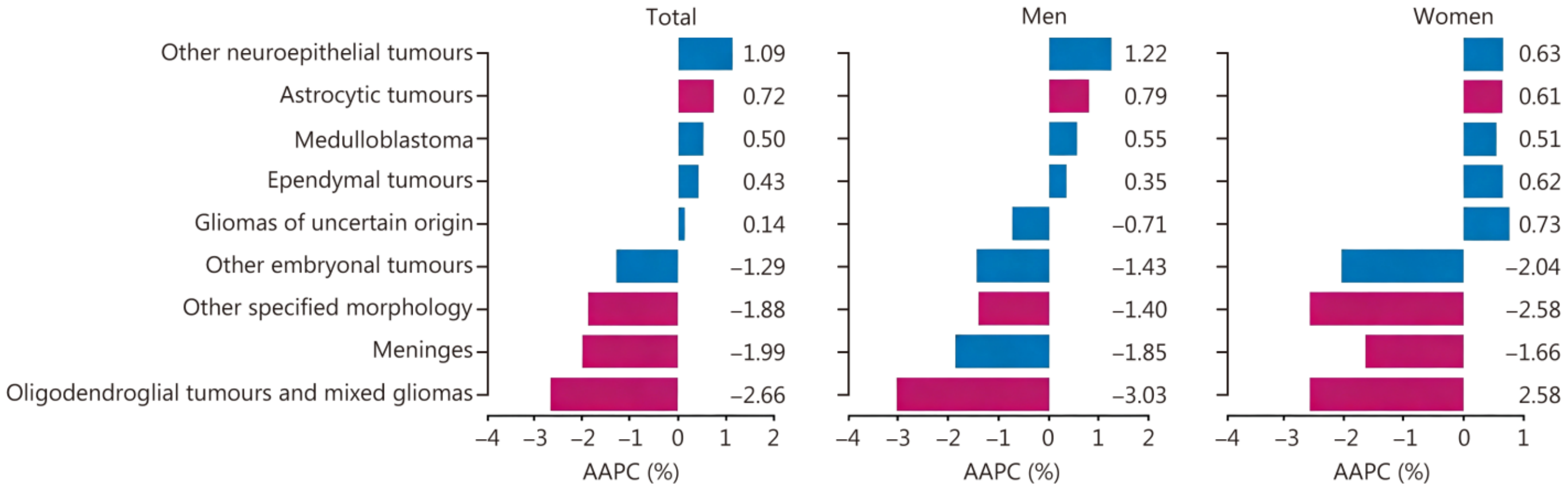

Fig. S23 The average annual percent change (AAPC) in age-standardized incidence of brain and CNS tumors by subtype, 2003–2017. Red bars represent AAPC with a statistically significant trend ($P<0.05$). Navy-blue bars represent AAPC is not statistically significant trend ($P\geq0.05$). CNS. Central nervous system

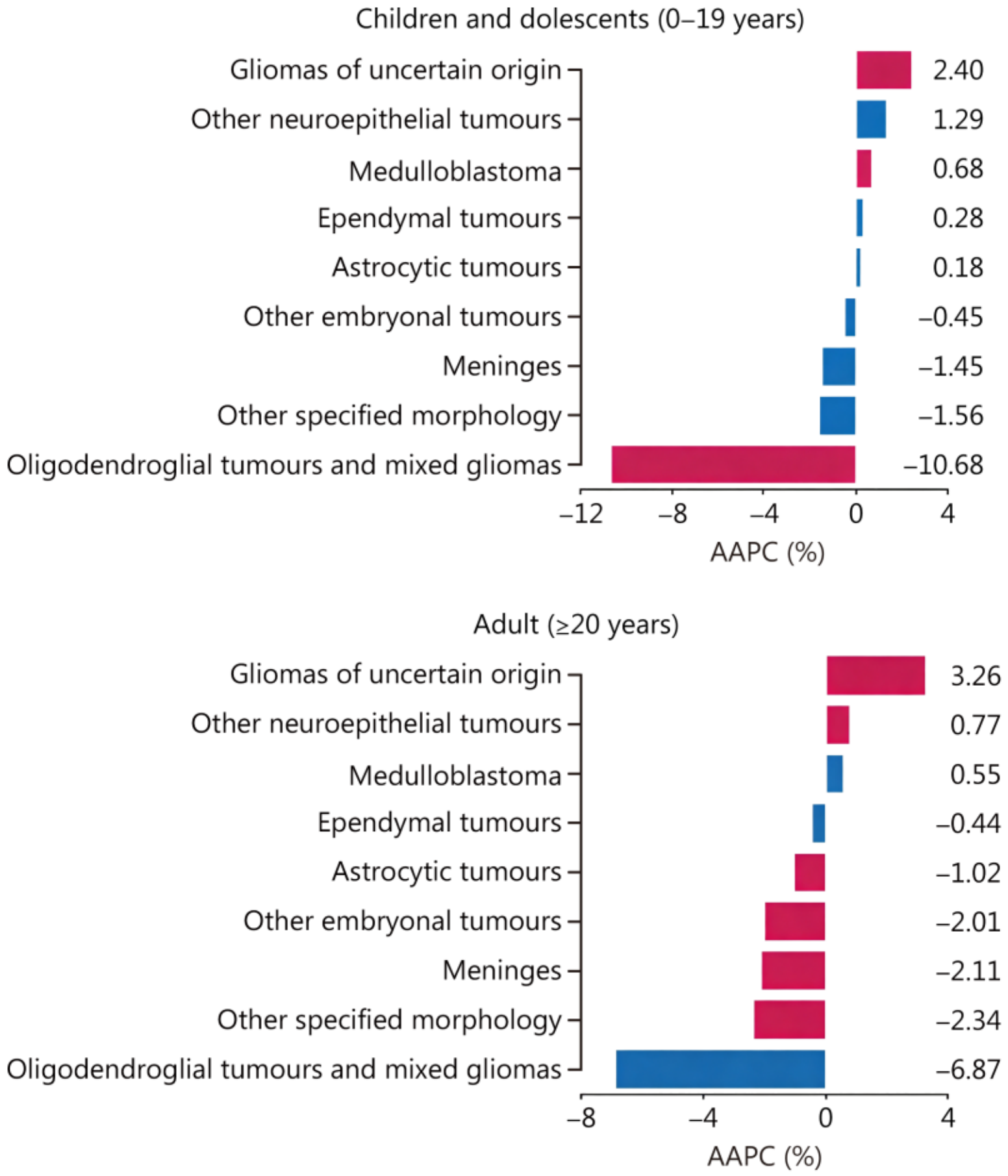

Fig. S24 The average annual percent change (AAPC) in age-standardized incidence of brain and CNS tumors by subtype and age group, 2003–2017. Red bars represent AAPC with a statistically significant trend (P<0.05). Navy-blue bars represent AAPC is not statistically significant trend (P≥0.05). CNS. Central nervous system

HDI level	ASIR	*RR* (95% CI)	*P*-value
Both sexes			
Very high HDI	4.69	Reference	
High HDI	3.80	0.68(0.55–0.85)	0.0005
Medium HDI	2.03	0.45(0.33–0.61)	<0.0001
Low HDI	1.97	0.31(0.22–0.43)	<0.0001
Men			
Very high HDI	5.43	Reference	
High HDI	3.94	0.69(0.55–0.86)	0.0010
Medium HDI	2.30	0.41(0.30–0.56)	<0.0001
Low HDI	2.45	0.30(0.21–0.42)	<0.0001
Women			
Very high HDI	4.00	Reference	
High HDI	3.66	0.67(0.53–0.85)	0.0008
Medium HDI	1.75	0.52(0.38–0.71)	<0.0001
Low HDI	1.53	0.32(0.22–0.45)	<0.0001

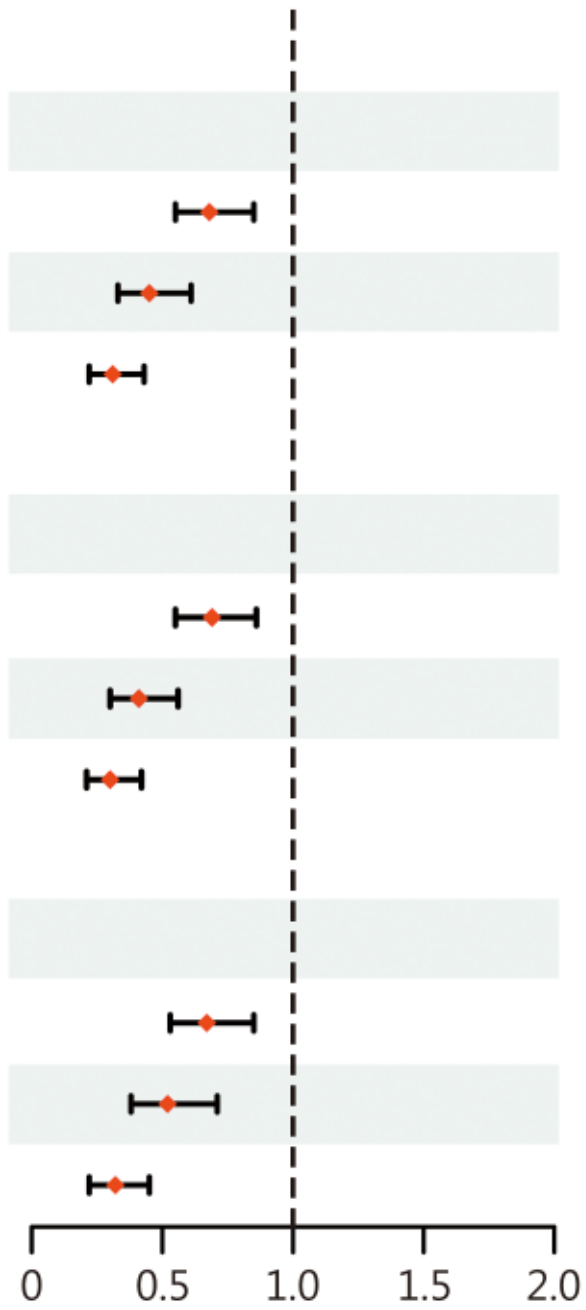

Fig. S25 The association between human development index (HDI) and ASIR of brain and CNS tumors in 186 countries and territories, 2024. Multivariable models were adjusted for Current Health Expenditure (CHE) as a percentage of Gross Domestic Product (GDP), CHE per capita in US$, population density, percentage of population living in urban areas. *RR*. Risk ratio; CI. Confidence interval; CNS. Central nervous system; ASIR. Age-standardized incidence rate

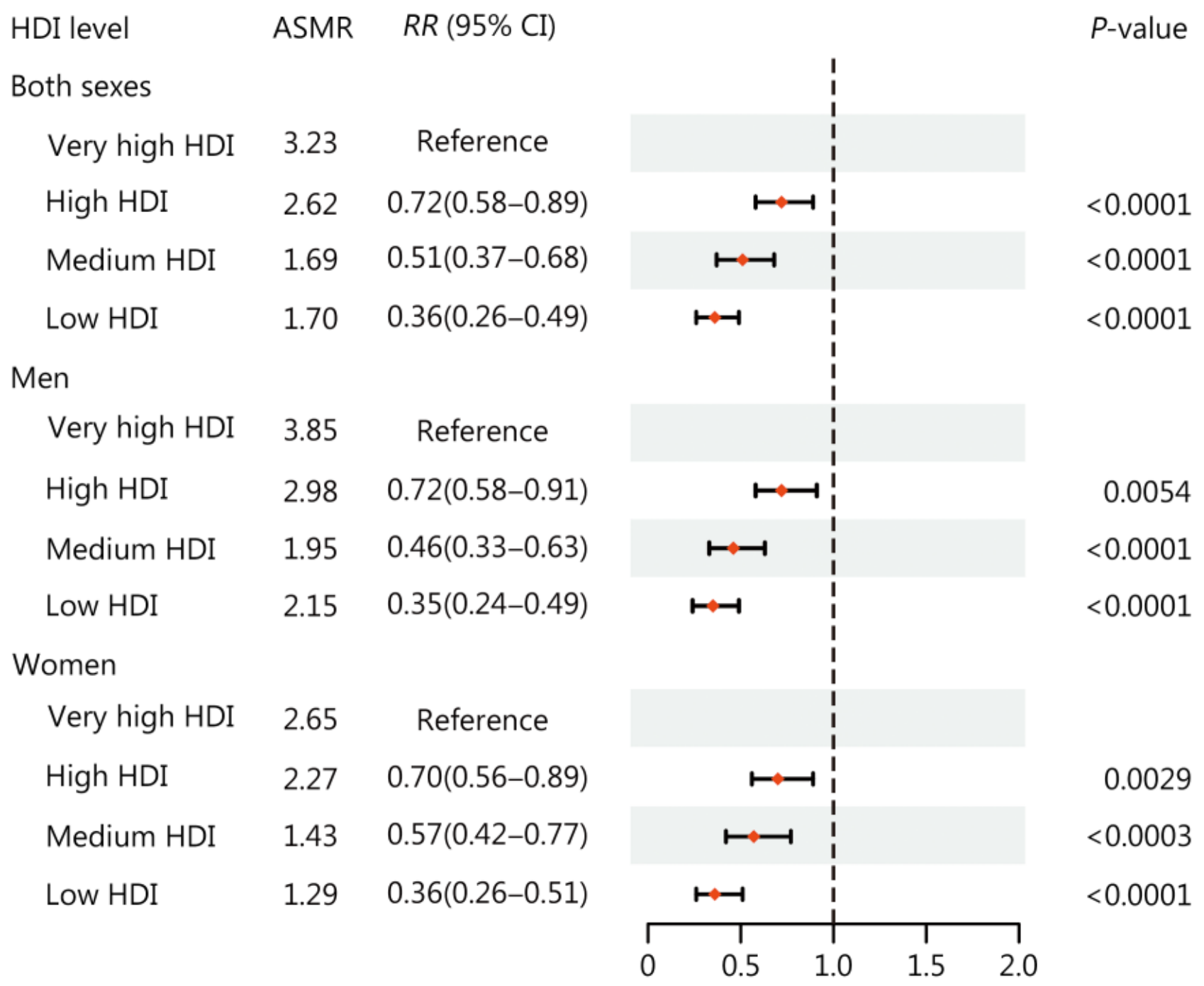

Fig. S26 The association between human development index (HDI) and ASMR of brain and CNS tumors in 186 countries and territories, 2024. Multivariable models were adjusted for Current Health Expenditure (CHE) as a percentage of Gross Domestic Product (GDP), CHE per capita in US$, population density, percentage of population living in urban areas. *RR*. Risk ratio; CI. Confidence interval; CNS. Central nervous system; ASMR. Age-standardized mortality rate

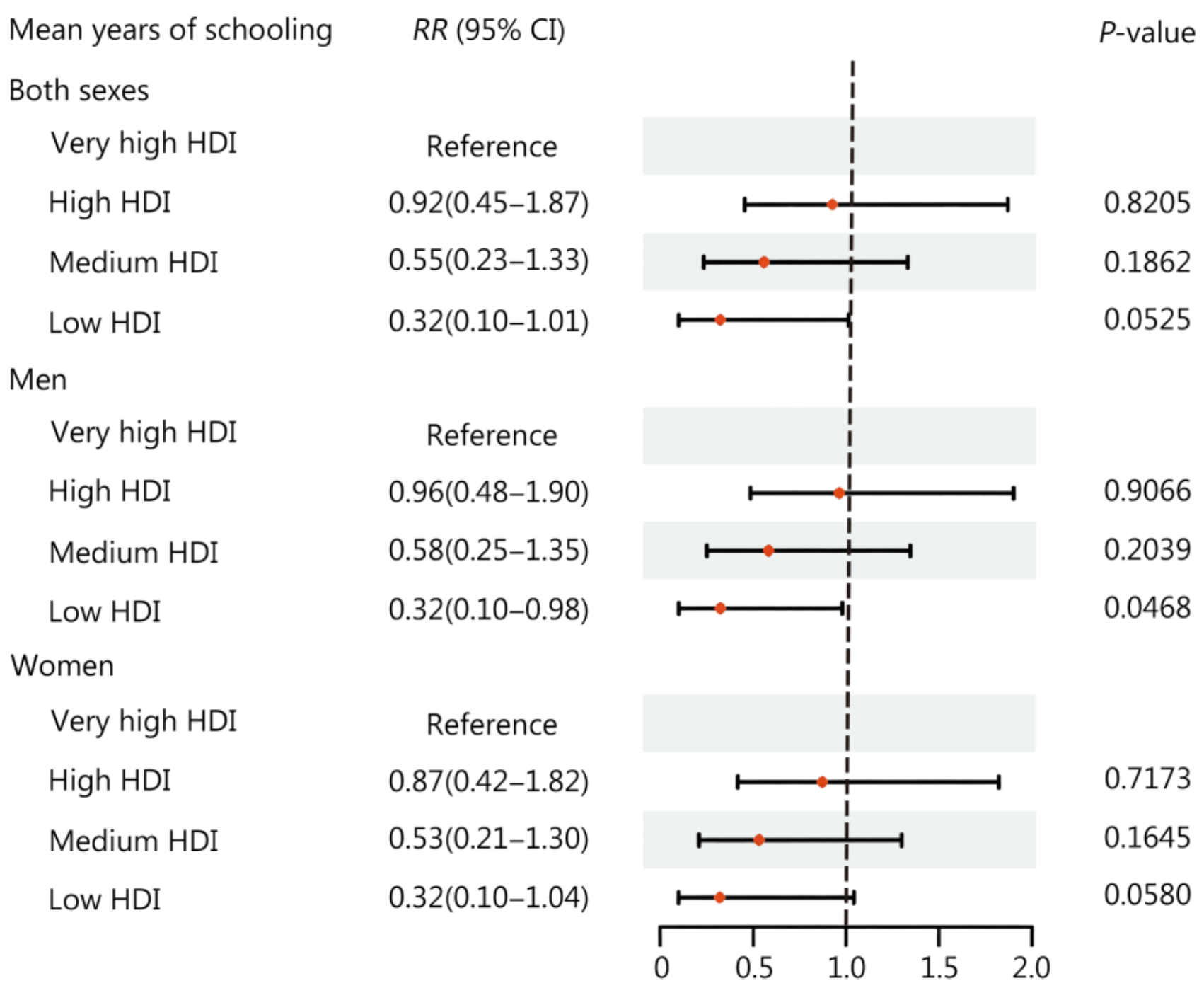

Fig. S27 The association between mean years of schooling and lifetime risk of developing from brain and CNS tumors in 186 countries and territories, 2024. Multivariable models were adjusted for Current Health Expenditure (CHE) as a percentage of Gross Domestic Product (GDP), CHE per capita in United States Dollar (USD), population density, percentage of population living in urban areas. *RR*. Risk ratio; CI. Confidence interval; HDI. Human development index; CNS. Central nervous system

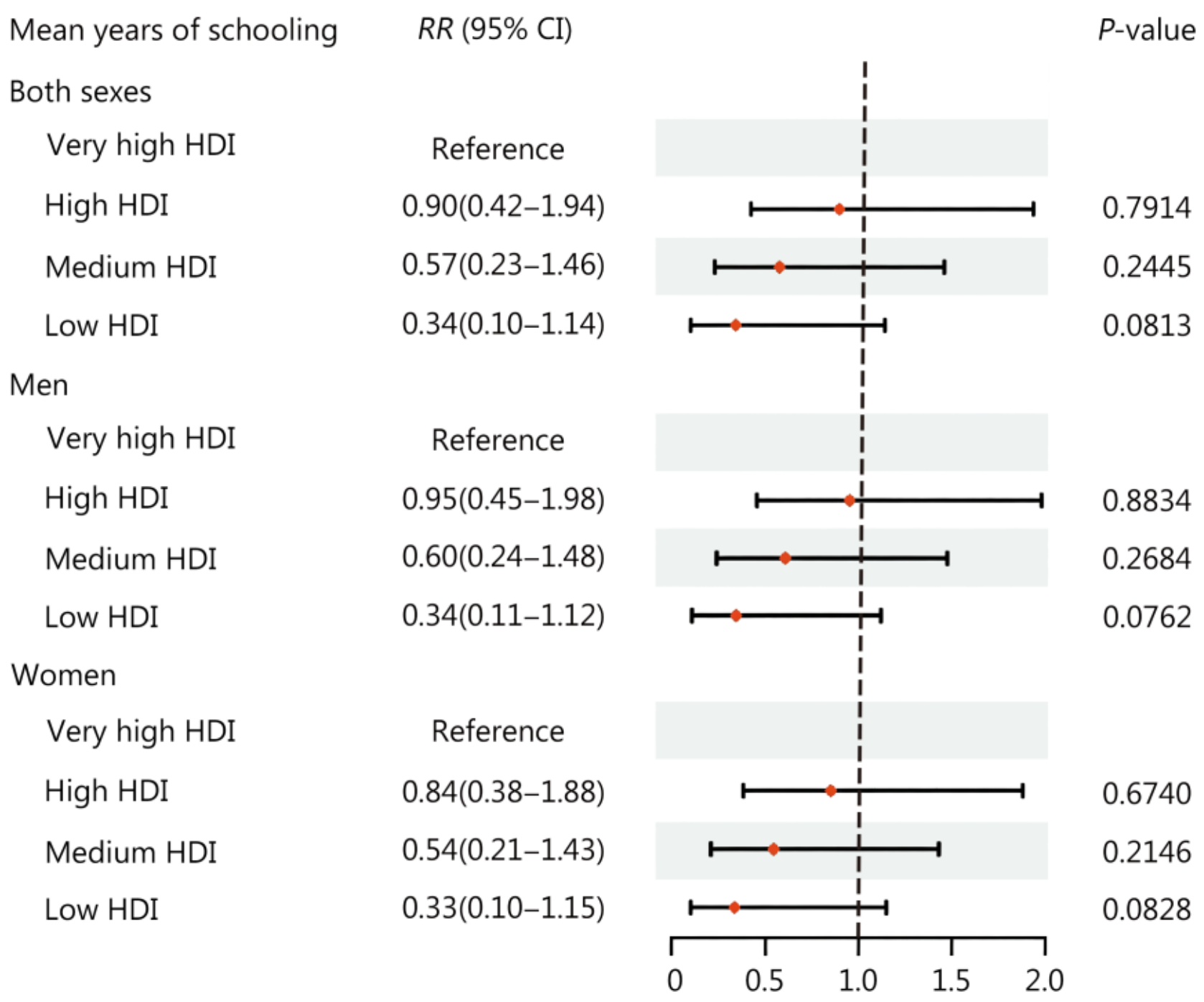

Fig. S28 The association between mean years of schooling and lifetime risk of dying from brain and CNS tumors in 186 countries and territories, 2024. Multivariable models were adjusted for Current Health Expenditure (CHE) as a percentage of Gross Domestic Product (GDP), CHE per capita in United States Dollar (USD), population density, percentage of population living in urban areas. *RR*. Risk ratio; CI. Confidence interval; HDI. Human development index; CNS. Central nervous system

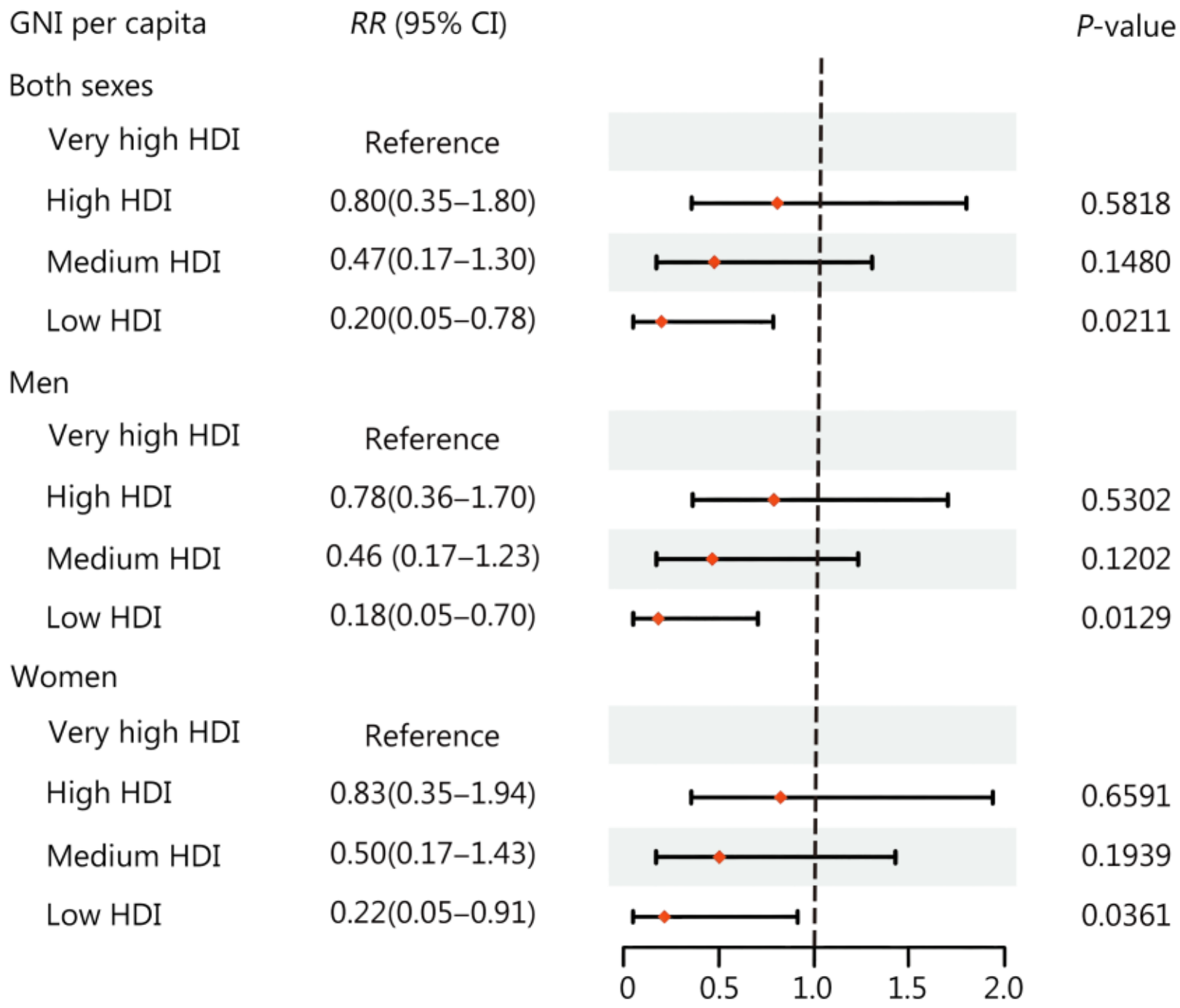

Fig. S29 The association between gross national income (GNI) per capita and lifetime risk of developing from brain and CNS tumors in 186 countries and territories, 2024. Multivariable models were adjusted for Current Health Expenditure (CHE) as a percentage of Gross Domestic Product (GDP), CHE per capita in United States Dollar (USD), population density, percentage of population living in urban areas. *RR*. Risk ratio; CI. Confidence interval; HDI. Human development index; CNS. Central nervous system

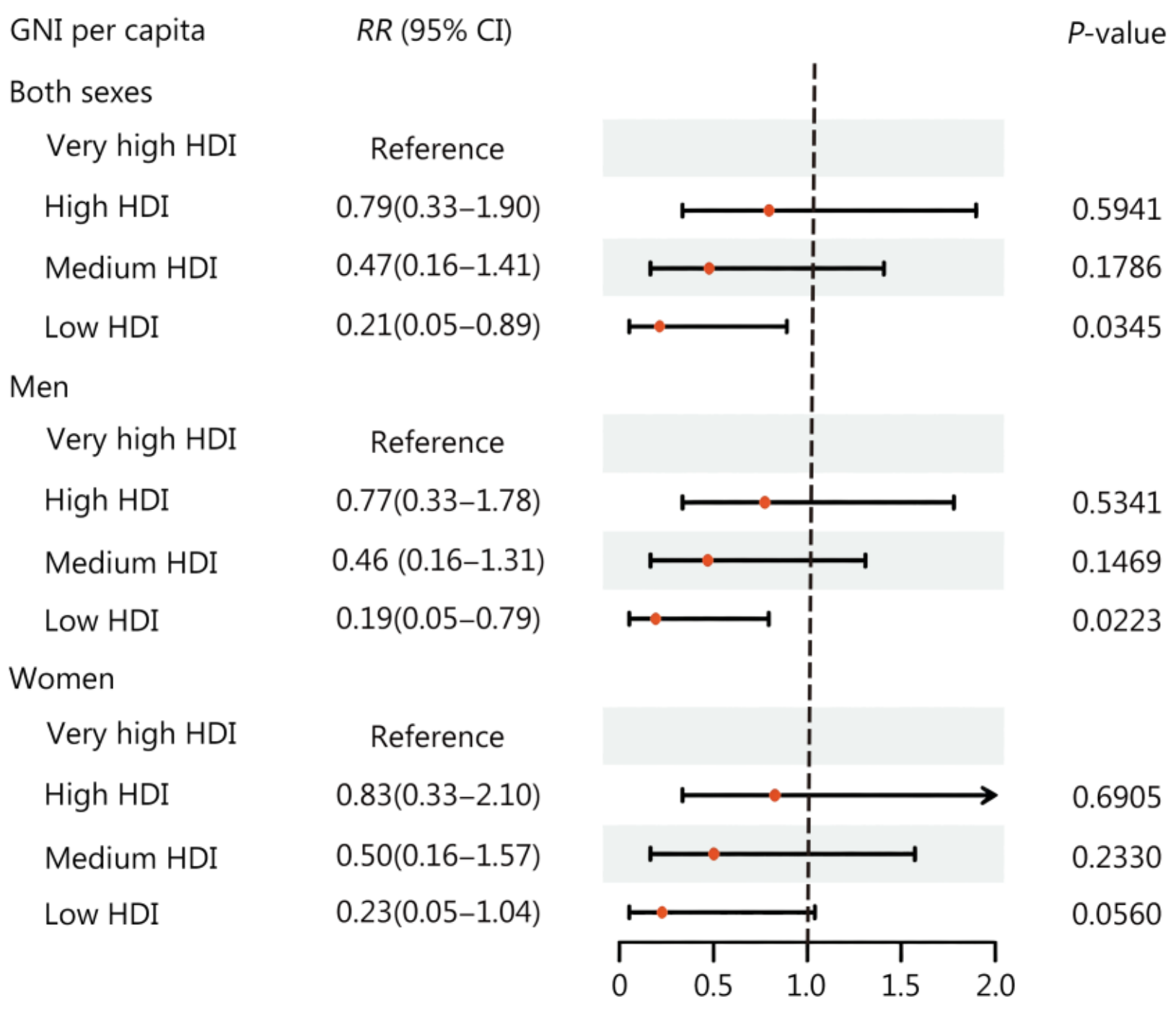

Fig. S30 The association between gross national income (GNI) per capita and lifetime risk of dying from brain and CNS tumors in 186 countries and territories, 2024. Multivariable models were adjusted for Current Health Expenditure (CHE) as a percentage of Gross Domestic Product (GDP), CHE per capita in United States Dollar (USD), population density, percentage of population living in urban areas. *RR*. Risk ratio; CI. Confidence interval; HDI. Human development index; CNS. Central nervous system

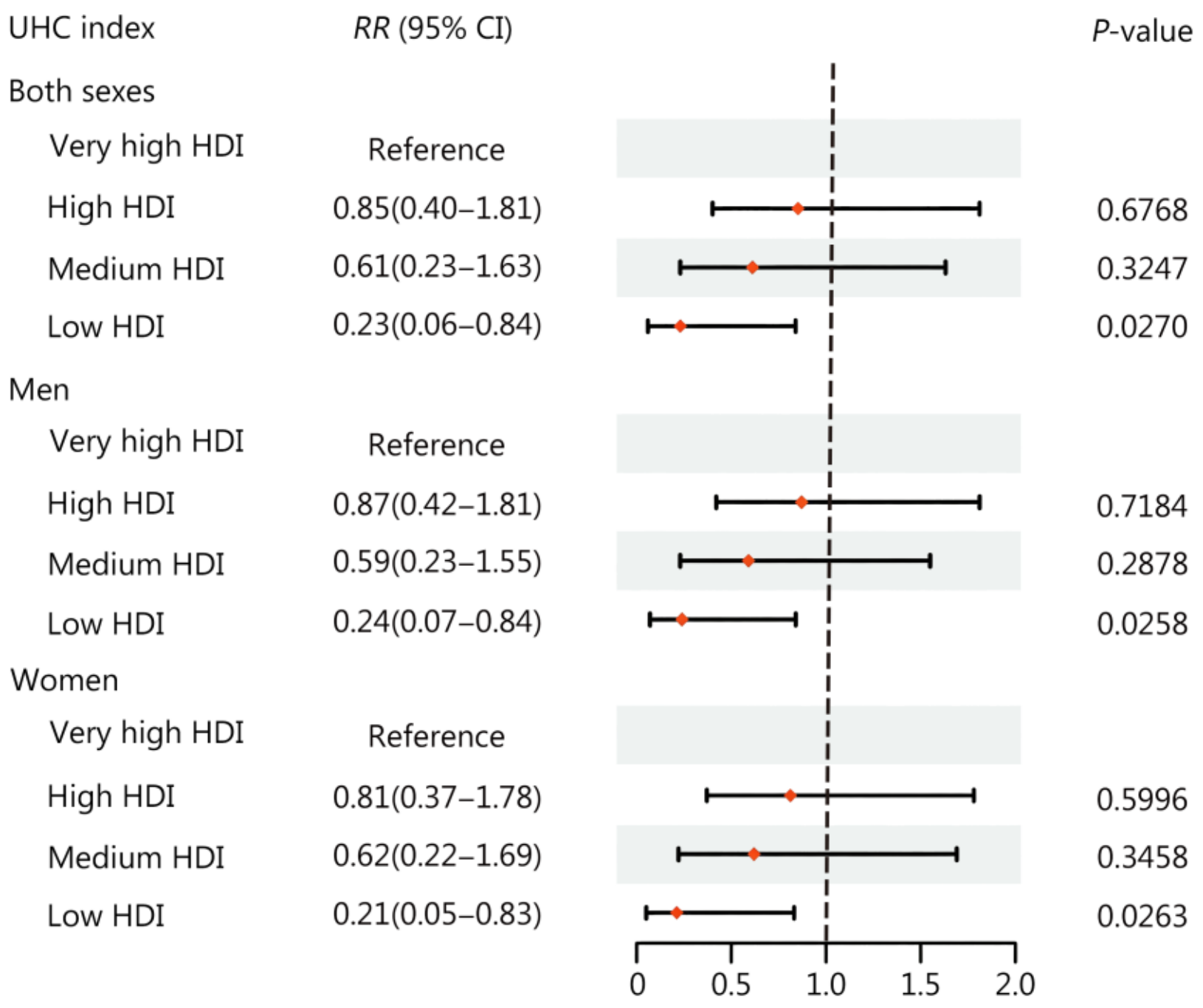

Fig. S31 The association between universal health coverage (UHC) index and lifetime risk of developing from brain and CNS tumors in 186 countries and territories, 2024. Multivariable models were adjusted for Current Health Expenditure (CHE) as a percentage of Gross Domestic Product (GDP), CHE per capita in United States Dollar (USD), population density, percentage of population living in urban areas. *RR*. Risk ratio; CI. Confidence interval; HDI. Human development index; CNS. Central nervous system

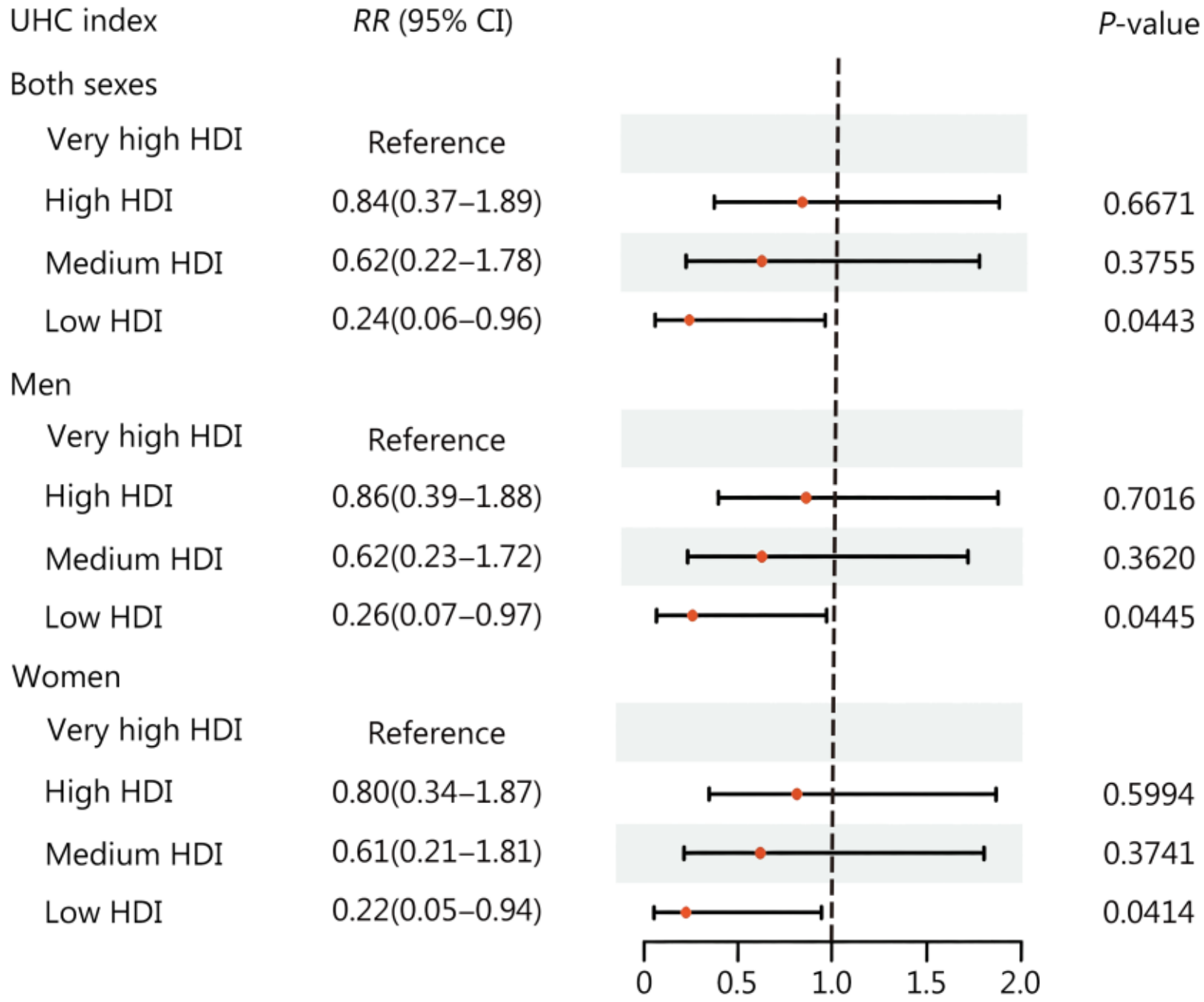

Fig. S32 The association between universal health coverage (UHC) index and lifetime risk of dying from brain and CNS tumors in 186 countries and territories, 2024. Multivariable models were adjusted for Current Health Expenditure (CHE) as a percentage of Gross Domestic Product (GDP), CHE per capita in United States Dollar (USD), population density, percentage of population living in urban areas. *RR*. Risk ratio; CI. Confidence interval; HDI. Human development index; CNS. Central nervous system

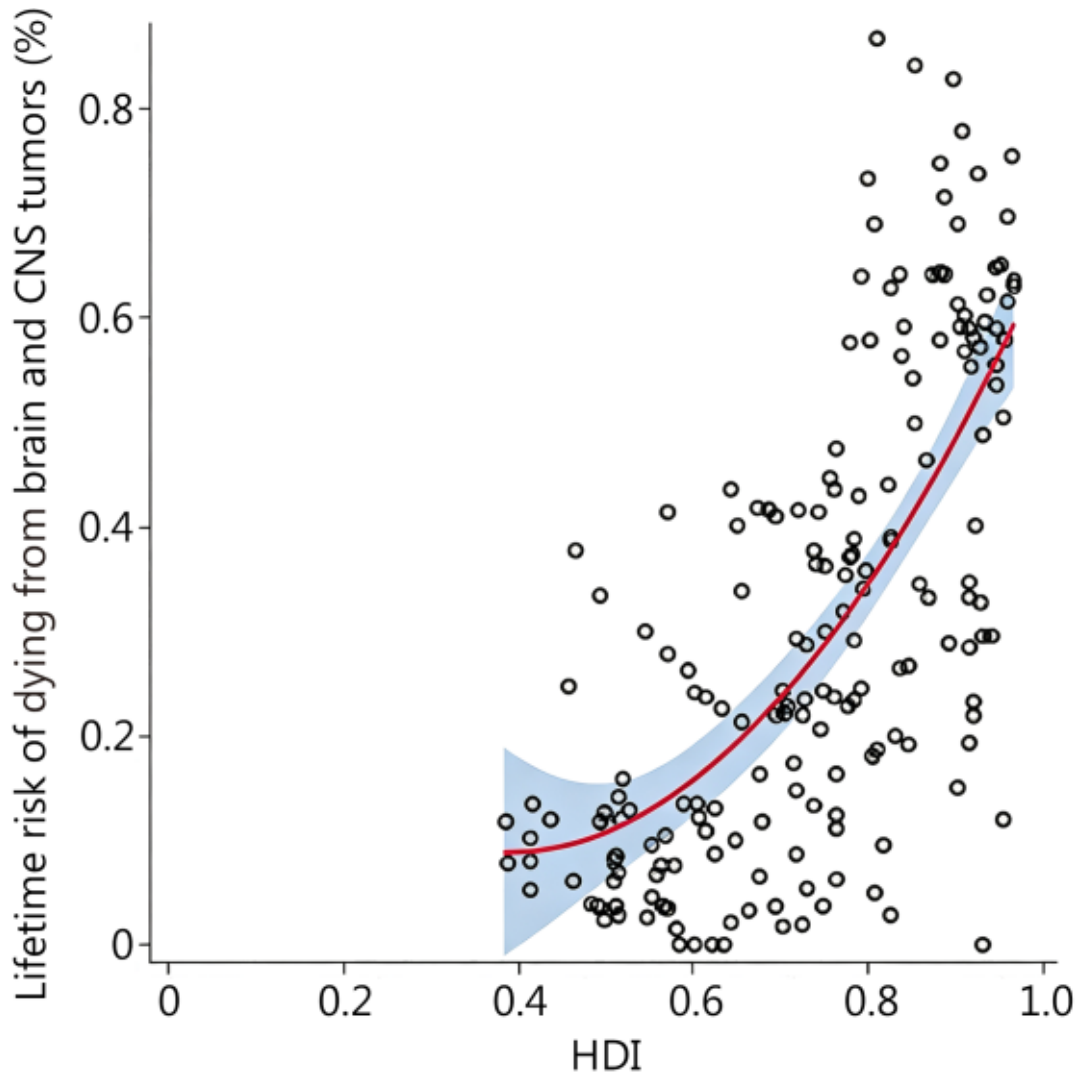

Fig. S33 Correlation between lifetime risk of dying from brain and CNS tumors and human development index (HDI) in both sexes. Red line was fitted with quadratic regression and 95% confidence interval. CNS. Central nervous system

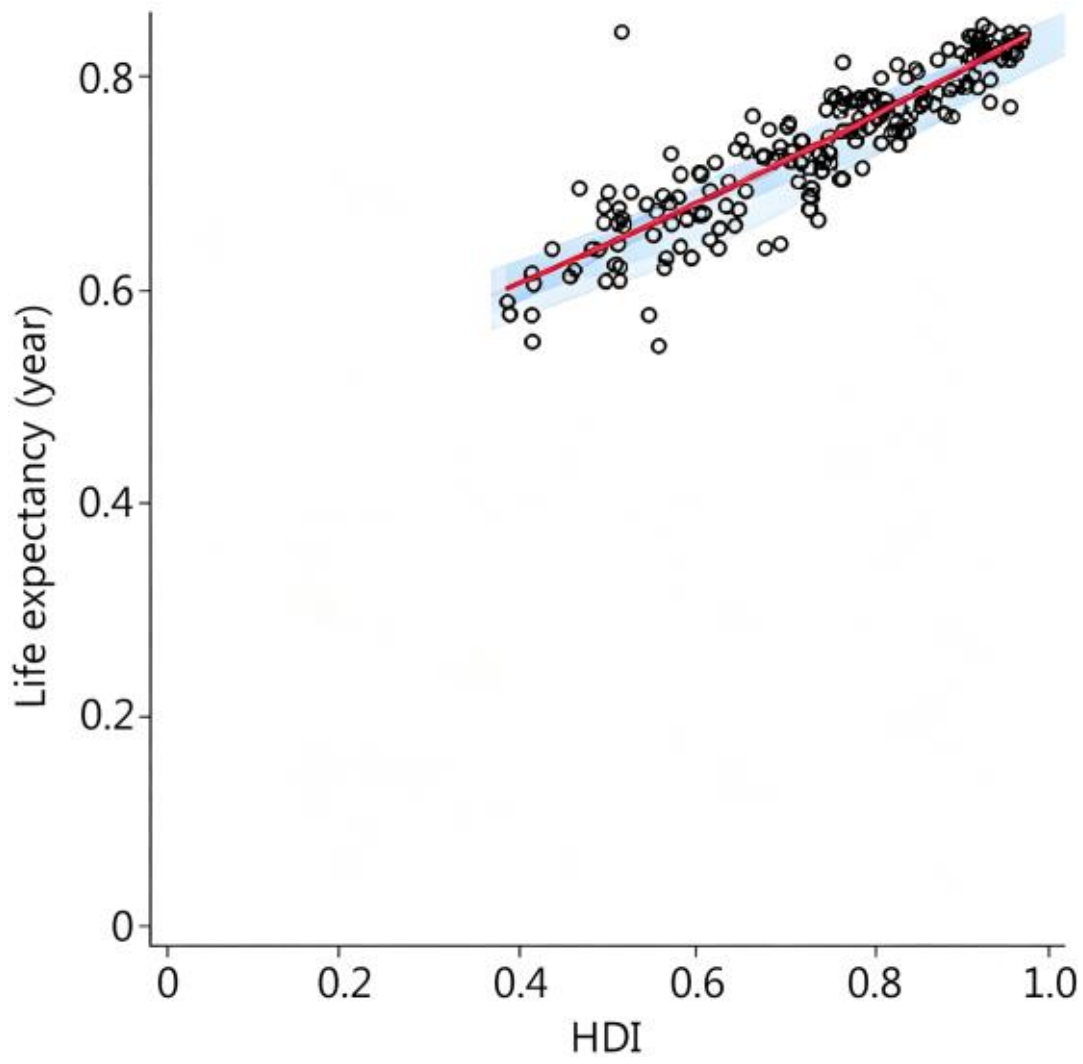

Fig. S34 Correlation between human development index (HDI) and life expectancy in both sexes. Red line was fitted with quadratic regression and 95% confidence interval. CNS. Central nervous system
